# Supplementary material for: An In Vivo Requirement for the Mediator Subunit Med14 in the Maintenance of Stem Cell Populations
Source: Stem Cell Reports. 2015 Mar 12;4(4):670–84. doi: 10.1016/j.stemcr.2015.02.006 (PMC4400641; doi:10.1016/j.stemcr.2015.02.006)
Supplement: Document S1. Supplemental Experimental Procedures and Figures S1 and S2 [file mmc1.pdf]

Stem Cell Reports

Supplemental Information

An In Vivo Requirement for the Mediator Subunit Med14 in the Maintenance of Stem Cell Populations

Jeffrey T.A. Burrows, Bret J. Pearson, and Ian C. Scott

Supplemental Figure 1

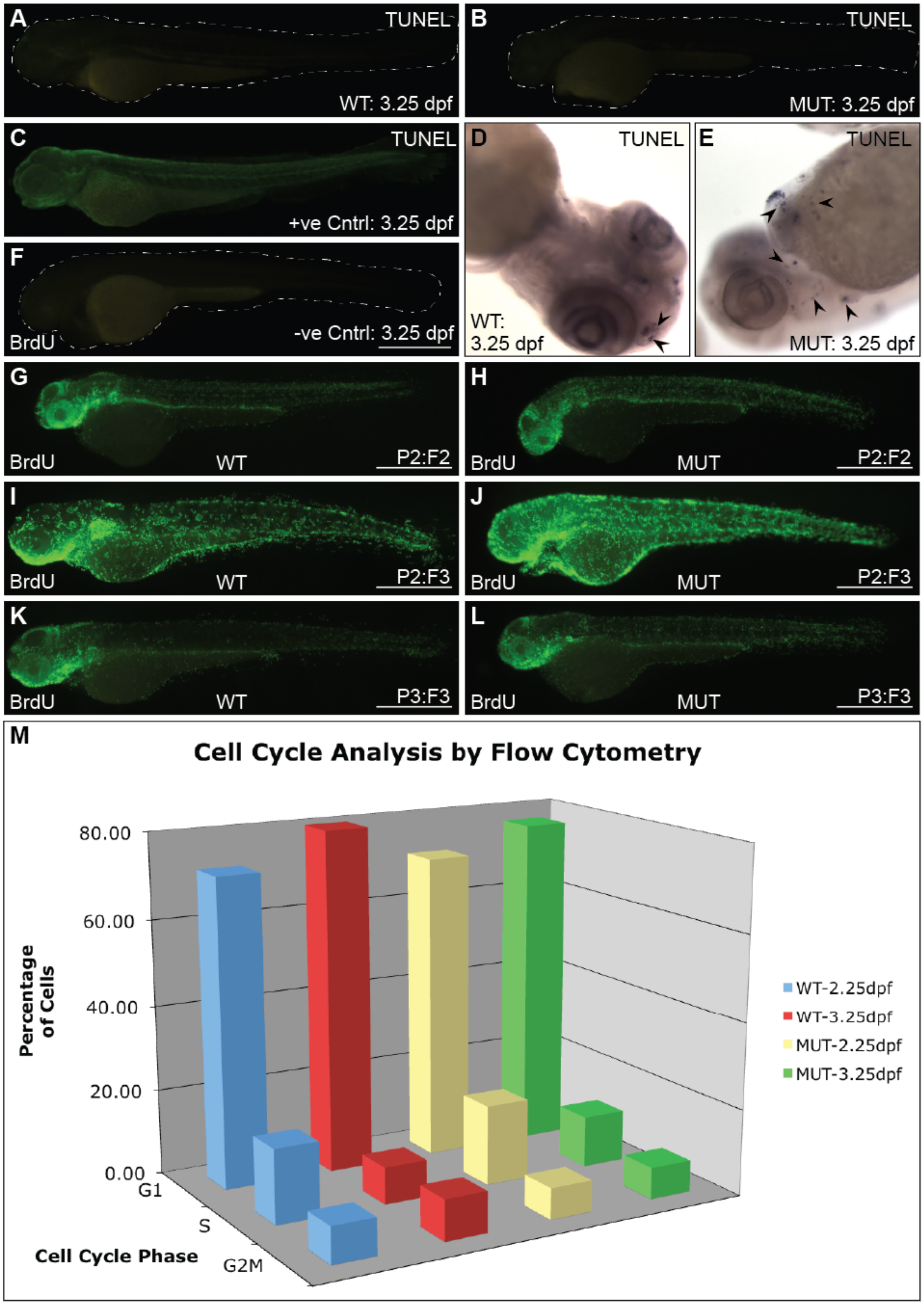

Supplemental Figure 1 (related to Figure 1): Cell Death, Cell Proliferation and Cell Cycle Parameters are Largely Normal in *log* Mutants.

(A-E) TUNEL assay for cell death in 3.25dpf embryos (embryos outlined in A and B). No TUNEL-positive cells were observed in wildtype or mutant embryos. Robust labeling of cell nuclei was evident in the positive control (DNase treated 3.25 wildtype embryos, C). (D-E) AP (Alkaline Phosphatase) amplification of signal revealed some TUNEL-positive cells in 3.25dpf wildtype and mutant embryos (black arrowheads). (F-L) Embryos were pulsed with BrdU for 10 minutes at 2dpf and fixed either immediately (P2F2) or after a 24-hour chase period (P2F3). A BrdU pulse and subsequent fix at 3dpf was also carried out (P3F3). (M) Results of cell cycle analysis by flow cytometry for DNA content on dissociated wildtype and mutant embryos. Percentage values are as follows (mean +/- std error for G1, S, G2M respectively): 2.25dpf WT (blue bars) (71.95 +/- 2.77, 17.28 +/- 2.74, 8.4 +/- 0.75), 3.25dpf WT (red bars) (79.98 +/- 2.05, 8.59 +/- 0.97, 9.42 +/- 0.36), 2.25dpf MUT (yellow bars) (71.28 +/- 0.78, 18.58 +/- 0.56, 7.20 +/- 0.30), 3.25 MUT (green bars) (77.05 +/- 1.54, 11.84 +/- 1.40, 7.31 +/- 0.30). Note that no significant difference is observed between comparisons between matched cell cycle phases for wildtype and mutant at 2.25dpf (p=0.82, 0.66, 0.17 for WT G1 vs. MUT G1, WT S vs. MUT S and WT G2M vs. MUT G2M respectively) and that changes in percentage of cells in each stage of the cell cycle follow the same trend in wildtype and mutant embryos when going from 2.25 to 3.25dpf (n = 3 biological replicates, 50 embryos per sample). Scale bars in (F-L) represent 0.5 mm.

Supplemental Figure 2

SUBUNIT	*med8*	*med17*	*med19*	*med1*	*med7*	*med31*	*med14*	*med23*	*med27*	*med12*	*CDK8*	*Cyclin C*
DOMAIN	Head	Head	Head	Middle	Middle	Middle	Tail	Tail	Tail	Kinase	Kinase	Kinase
SC DEFECT	No	Yes	Yes	No	Yes	No	Yes	No	Yes	Yes	No	No

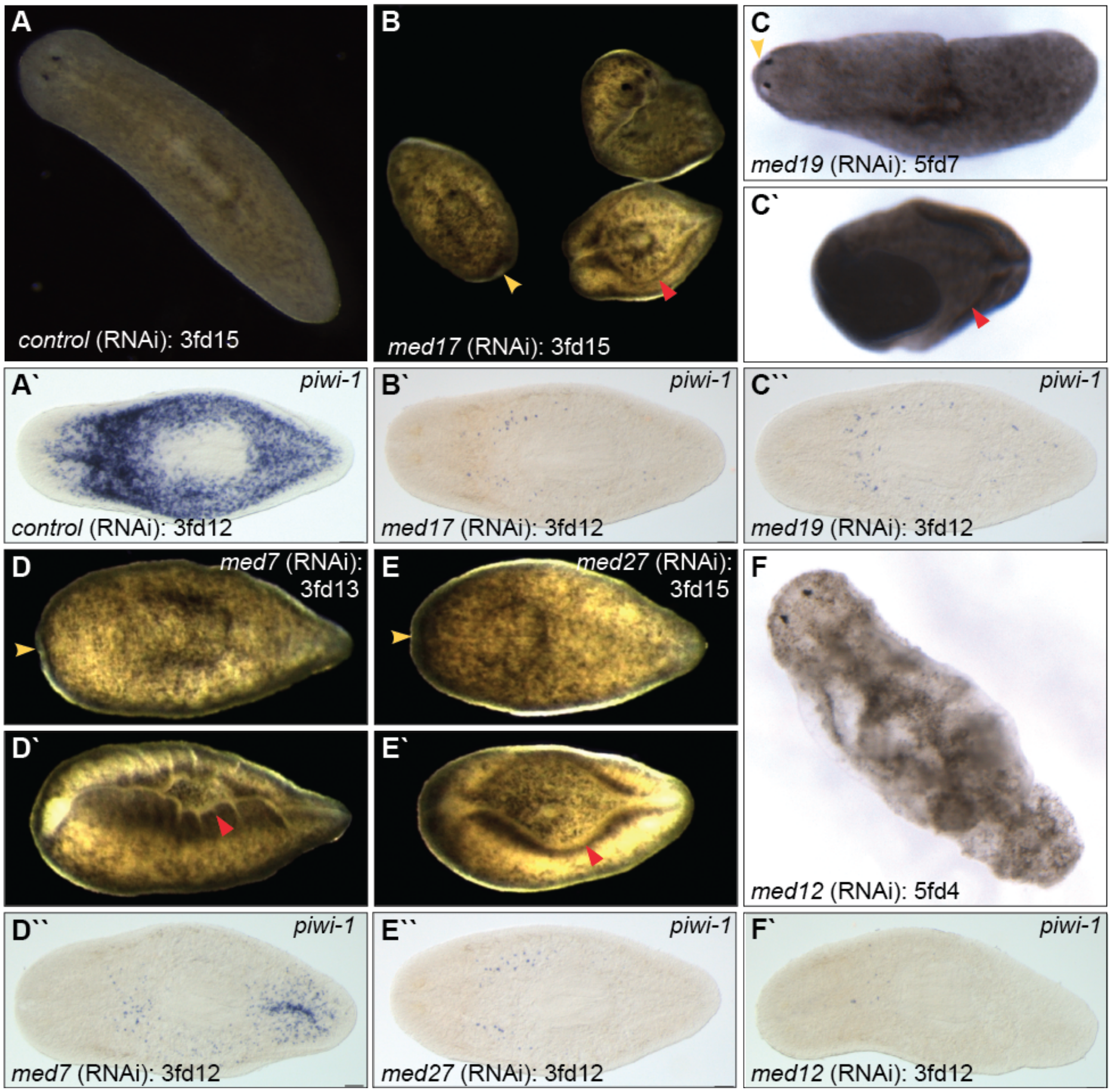

Supplemental Figure 2 (related to Figure 6): Multiple Mediator Components in Planarians are Required for Stem Cell Maintenance.

Table - A stem cell (SC) defect was observed for highlighted subunits. (A-F`) Results of a screen aimed to determine whether the requirement for stem cell maintenance is

specific to *med14* or a general feature of Mediator Complex. (A, A`) Representative live and *piwi-1* ISH image of a control RNAi animal, respectively. (B, B`) Representative *med17* (RNAi) planarian at displaying a stem cell defect phenotype. Note head regression (yellow arrowhead) and ventral curling (red arrowhead, B) as well as loss of *piwi-1* expression (B`). Dorsal and ventral views (C, C`) respectively of an early stem cell defect phenotype observed in representative *med19* (RNAi) animal. (C``) ISH for *piwi-1* confirms loss of the stem cell population in *med19* (RNAi) planaria. (D, D`) Dorsal and ventral views respectively of a representative *med7* (RNAi) animal displaying a stem cell defect phenotype. (D``) A varying degree of *piwi-1* expression loss was observed by ISH in *med7* (RNAi) planaria ranging from sporadic patches to complete absence. (E, E`) Dorsal and ventral views, respectively, of a representative *med27* (RNAi) animal. (E``) ISH for *piwi-1* confirms loss of the stem cell population following *med27* RNAi. (F) Live dorsal view of a representative *med12* (RNAi) planarian displaying a lysis phenotype. (F`) *piwi-1* ISH image confirms loss of the stem cell population in a representative *med12* (RNAi) animal.

SUPPLEMENTAL EXPERIMENTAL PROCEDURES

Probes for RNA *in situ* Hybridization

Templates for zebrafish *med14* (ZDB-GENE-030131-8867), *opn1sw1* (ZDB-GENE-991109-25), *mz98* (*col15a1b,* ZDB-GENE- 070912-689), *cmyb* (ZDB-GENE- 991110-14), *lgr4* (ENSDARG00000060542) and *ltbp3* (ZDB-GENE-060526-130) riboprobes were generated by RT-PCR from 72hpf cDNA template using gene-specific primers and subcloned into pGEM-T Easy vector (Promega). Previously described riboprobe templates for *myl7* (*cmlc2*, ZDB-GENE-991019-3), *vmhc* (ZDB-GENE-991123-5) and *myh6* (*amhc*, ZDB-GENE-031112-1) were used (Yelon et al., 1999). Probes were made via *in vitro* transcription from linearized DNA templates using a DIG RNA Labeling Kit (Roche). The planarian *med14* homolog was found using BLAST against the assembled *S. mediterranea* genome (Robb et al., 2008). It was then cloned by RT-PCR from cDNA using gene-specific primers into pRT4P vector. The remaining planarian Mediator subunits (*med8*, *med17*, *med19*, *med1*, *med7*, *med31*, *med23*, *med27*, *med12*, *Cyclin C* and *CDK8*) were cloned in same manner. Riboprobe was generated by PCR amplification and reverse transcription using a DIG labeling mix. Additional planarian riboprobes used have been previously characterized (*smedwi-1/piwi-1* (Reddien et al., 2005), *prog-1* (Eisenhoffer et al., 2008) *pc2* and porcn (Gurley et al., 2008), *laminin* (Cebria and Newmark, 2007), *collagen* (Witchley et al., 2013), *cavii-1* (Rink et al., 2011), *ovo* (Lapan and Reddien, 2012), *pcna* (Orii et al., 2005) and *h2b* (Guo et al., 2006)).

Mean Normalized Expression Analysis

Mean Normalized Expression (MNE = [$E_{GFP\text{-}RNA}^{(GFP\text{-}RNA\ C_T)}$ / $E_{\beta\text{-}actin}^{(\beta\text{-}actin\ C_T)}$] / GCN) for *β-actin* was calculated using a universal reference approach which employs 2 exogenous spikes as pseudo-housekeeping genes for normalization of the gene expression (Bower et al., 2007). Briefly, GFP-RNA (10ng) and DNA (1.0ng) spikes were added to homogenized embryos prior to simultaneous extraction of RNA and DNA using a Qiagen AllPrep DNA/RNA Mini Kit (Qiagen). RNA samples were treated with DNase as part of the extraction protocol (RNase-Free DNase Set, Qiagen). Genome Copy Number (GCN = $E_{GFP\text{-}DNA}^{(GFP\text{-}DNA\ C_T)}$ / $E_{NC\text{-}DNA}^{(NC\text{-}DNA\ C_T)}$) was calculated from qPCR measurement of genomic copies of a conserved non-coding region (NC-DNA, SE: TCTCCGGACTCCACAACTT, AS: AACAGCTCGAGTGGATTGTG) (Shin et al., 2005) normalized to the GFP-DNA spike (SE: TGTTATGGTGTTCAATGCTTCTC, AS: TGACTTCAGCACGTGTCTTGT). Following RT-PCR of the RNA fractions, the expression of *β-actin* (using the same primers as previous stated) and the GFP-RNA (using the same primers for the GFP-DNA spike) were measured by qPCR and then adjusted by GCN. In all cases amplification Efficiency (E) was assessed through a dilution series. The DNA spike was generated through PCR amplification of the GFP coding sequence contained in a pCS2+ plasmid (SE: GTCAGTGGAGAGGGTGAAGG, AS: AAAGGGCAGATTGTGTGGAC). The RNA spike was generated off the same plasmid by *in vitro* transcription as described for the *med14* overexpression/rescue analysis.

SUPPLEMENTAL REFERENCES

Bower, N.I., Moser, R.J., Hill, J.R., and Lehnert, S.A. (2007). Universal reference method for real-time PCR gene expression analysis of preimplantation embryos. Biotechniques *42*, 199-206.

Cebria, F., and Newmark, P.A. (2007). Morphogenesis defects are associated with abnormal nervous system regeneration following roboA RNAi in planarians. Development *134*, 833-837.

Eisenhoffer, G.T., Kang, H., and Sanchez Alvarado, A. (2008). Molecular analysis of stem cells and their descendants during cell turnover and regeneration in the planarian Schmidtea mediterranea. Cell Stem Cell *3*, 327-339.

Guo, T., Peters, A.H., and Newmark, P.A. (2006). A Bruno-like gene is required for stem cell maintenance in planarians. Dev Cell *11*, 159-169.

Gurley, K.A., Rink, J.C., and Sanchez Alvarado, A. (2008). Beta-catenin defines head versus tail identity during planarian regeneration and homeostasis. Science *319*, 323-327.

Lapan, S.W., and Reddien, P.W. (2012). Transcriptome analysis of the planarian eye identifies ovo as a specific regulator of eye regeneration. Cell Rep *2*, 294-307.

Orii, H., Sakurai, T., and Watanabe, K. (2005). Distribution of the stem cells (neoblasts) in the planarian Dugesia japonica. Dev Genes Evol *215*, 143-157.

Reddien, P.W., Oviedo, N.J., Jennings, J.R., Jenkin, J.C., and Sanchez Alvarado, A. (2005). SMEDWI-2 is a PIWI-like protein that regulates planarian stem cells. Science *310*, 1327-1330.

Rink, J.C., Vu, H.T., and Sanchez Alvarado, A. (2011). The maintenance and regeneration of the planarian excretory system are regulated by EGFR signaling. Development *138*, 3769-3780.

Robb, S.M., Ross, E., and Sanchez Alvarado, A. (2008). SmedGD: the Schmidtea mediterranea genome database. Nucleic Acids Res *36*, D599-606.

Shin, J.T., Priest, J.R., Ovcharenko, I., Ronco, A., Moore, R.K., Burns, C.G., and MacRae, C.A. (2005). Human-zebrafish non-coding conserved elements act in vivo to regulate transcription. Nucleic Acids Res *33*, 5437-5445.

Witchley, J.N., Mayer, M., Wagner, D.E., Owen, J.H., and Reddien, P.W. (2013). Muscle cells provide instructions for planarian regeneration. Cell Rep *4*, 633-641.

Yelon, D., Horne, S.A., and Stainier, D.Y. (1999). Restricted expression of cardiac myosin genes reveals regulated aspects of heart tube assembly in zebrafish. Dev Biol *214*, 23-37.
