# Supplementary material for: An In Vivo Requirement for the Mediator Subunit Med14 in the Maintenance of Stem Cell Populations
Source: Stem Cell Reports. 2015 Mar 12;4(4):670–84. doi: 10.1016/j.stemcr.2015.02.006 (PMC4400641; doi:10.1016/j.stemcr.2015.02.006)
Supplement: Document S2. Article plus Supplemental Information [file mmc2.pdf]

Stem Cell Reports
Article
OPEN ACCESS

An In Vivo Requirement for the Mediator Subunit Med14 in the Maintenance of Stem Cell Populations

Jeffrey T.A. Burrows,[1,4] Bret J. Pearson,[1,2,4] and Ian C. Scott[1,3,4,*]
[1]Department of Molecular Genetics, University of Toronto, Toronto, ON M5S 1A8, Canada
[2]Ontario Institute for Cancer Research, Toronto, ON M5G 0A3, Canada
[3]Richard Lewar Centre for Excellence in Cardiovascular Research, Toronto, ON M5G 1L7, Canada
[4]Program in Developmental and Stem Cell Biology, The Hospital for Sick Children, Toronto, ON M5G 0A4, Canada
*Correspondence: ian.scott@sickkids.ca
http://dx.doi.org/10.1016/j.stemcr.2015.02.006
This is an open access article under the CC BY license (http://creativecommons.org/licenses/by/4.0/).

SUMMARY

The Mediator complex has recently been shown to be a key player in the maintenance of embryonic and induced pluripotent stem cells. However, the in vivo consequences of loss of many Mediator subunits are unknown. We identified *med14* as the gene affected in the zebrafish *logelei* (*log*) mutant, which displayed a morphological arrest by 2 days of development. Surprisingly, microarray analysis showed that transcription was not broadly affected in *log* mutants. Indeed, *log* cells transplanted into a wild-type environment were able to survive into adulthood. In planarians, RNAi knockdown demonstrated a requirement for *med14* and many other Mediator components in adult stem cell maintenance and regeneration. Multiple stem/progenitor cell populations were observed to be reduced or absent in zebrafish *med14* mutant embryos. Taken together, our results show a critical, evolutionarily conserved, in vivo function for Med14 (and Mediator) in stem cell maintenance, distinct from a general role in transcription.

INTRODUCTION

First identified in yeast (Kelleher et al., 1990; Thompson et al., 1993), the core Mediator complex consists of three distinct modules (Head, Middle, and Tail), with a fourth Kinase module present in some cases. Mediator physically links enhancer bound regulatory factors to RNA polymerase II (Pol II) through context-specific interactions with its Tail and Head subunits, respectively (Hengartner et al., 1995; Kim et al., 1994; Thompson et al., 1993). Work in yeast suggesting that Mediator is present at the promoters of nearly all protein coding genes and is required for both basal and activator-mediated transcription (Holstege et al., 1998; Thompson and Young, 1995) has led to the view that Mediator is part of the general transcription machinery; however, analysis of several Mediator mutants in plants and animals has not supported this model. Specific subunits have been shown to control only a subset of target genes that in turn affect specific developmental or organ-specific processes (reviewed in Hentges, 2011). The multitude of interactions documented for the 31 subunits of the Mediator complex delineate its vast functional versatility and has led to the more recent view of Mediator as an integrative hub of transcriptional regulation.

Development at a cellular level involves progression along a continuum from complete plasticity to terminal differentiation. For most cells, cell fate becomes "locked in" as development proceeds (Ho and Kimmel, 1993; Parameswaran and Tam, 1995). Stem and progenitor cells are capable of halting their progression along this developmental path and act as reserves for tissue homeostasis and regeneration. Much of what is known on how cells maintain their "stemness" has come from studying cultured embryonic stem cells (ESCs), which has revealed a complex network of transcription factors that act in concert to maintain pluripotency (Nichols et al., 1998; Takahashi and Yamanaka, 2006). Intriguingly, an RNAi screen for key regulators of pluripotency maintenance in mouse ESCs (Kagey et al., 2010) uncovered 12 subunits of Mediator, with the strongest effect resulting from knockdown of Med14. Med12 has additionally been shown to act together with Nanog to regulate a stem cell gene signature in mouse ESCs (Tutter et al., 2009). Whether the role of Mediator function in ESC maintenance extends generally to in vivo stem cell populations remains largely unknown.

In this study, we found that while zebrafish *med14* mutant embryos were largely arrested in development, there was a surprisingly limited effect on overall transcription. Transplantation experiments demonstrated that Med14 function is largely dispensable for cell survival into adulthood. Loss of *med14*, as well as several other Mediator components, in the planarian *Schmidtea mediterranea* resulted in severe stem cell and regeneration defects, with transcription in other tissues apparently unaffected. Examination of *med14* mutant zebrafish embryos also suggested a function in stem cell maintenance and regeneration. Taken together, our results show that Med14 has a conserved function in the maintenance of both embryonic and adult stem cell populations and suggest a broader in vivo role for Mediator in stem cell maintenance.

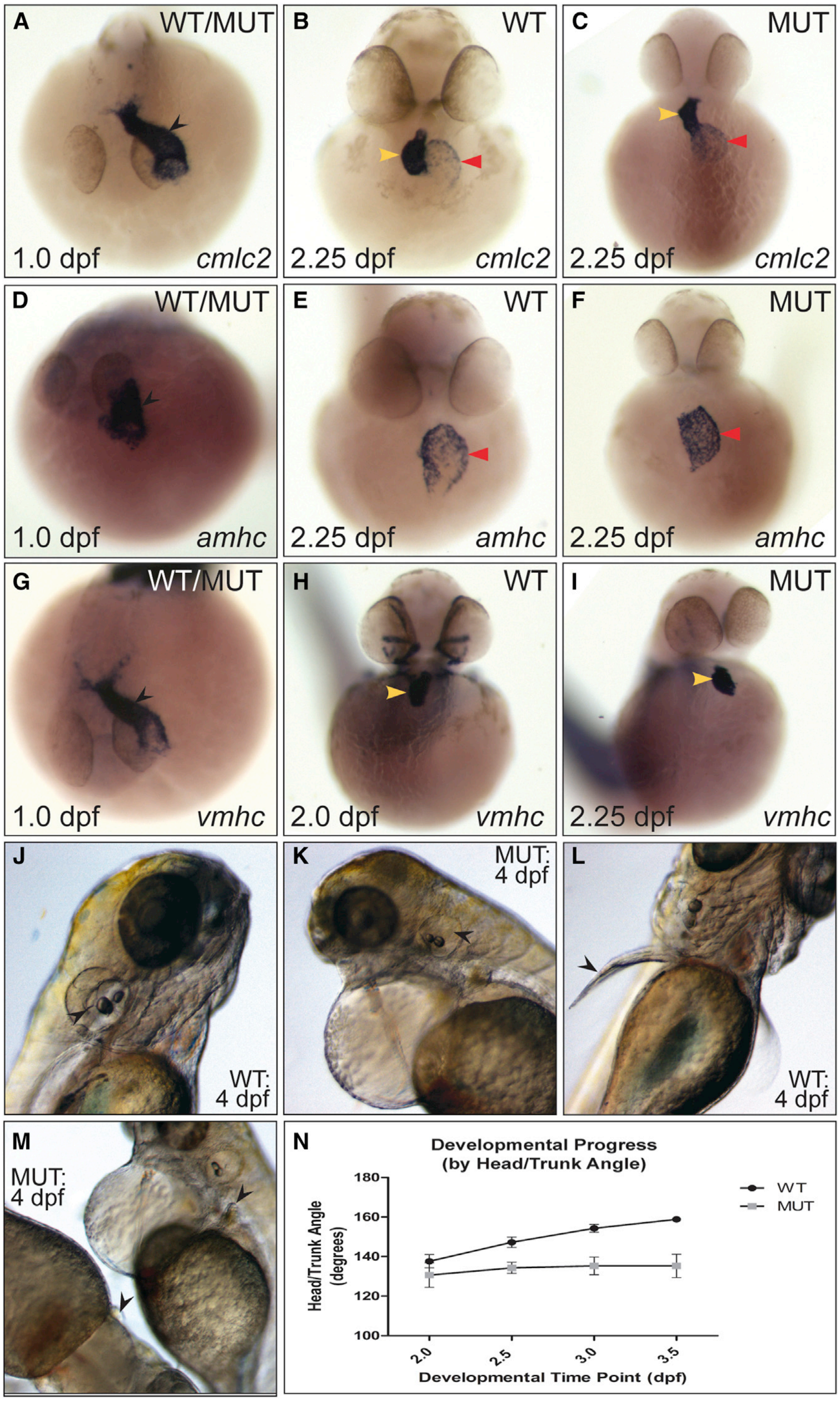

Figure 1. Characterization of the *log* Mutant Phenotype

(A–I) Expression of cardiac markers in (WT) and *log* mutant (MUT) embryos. While at 1.0 dpf the linear heart tube (black arrowhead) was apparent in all embryos ("WT/MUT"), at 2.25 dpf *log* mutant hearts were unlooped, with the ventricle directly above the atrium (yellow and red arrowheads, respectively).

(J–M) At 4.0 dpf, semicircular canals in the otic vesicles (black arrow heads in J and K) and elongation of the pectoral fins (black arrowheads in L and M) were absent in mutant embryos.

(N) Change in head/trunk angle over time in WT and mutant embryos (10 WT and 20 mutants measured per time point).

See also Figure S1.

RESULTS

Zebrafish *logelei* Mutants Have a Pleiotropic Phenotype Suggestive of Developmental Arrest

A novel (*s231*) allele of the *logelei* (*log*) mutant was previously isolated in a screen for mutations affecting cardiovascular development (Jin et al., 2007). At 1-day post-fertilization (dpf), *log* mutant hearts appeared completely normal (Figure 1A). Cardiac defects first became apparent in *log* mutants by 2 dpf, with a failure of heart looping (Figures 1B and 1C). By RNA in situ hybridization (ISH), expression of the chamber-specific markers *myh6*/*amhc* (atrium) and

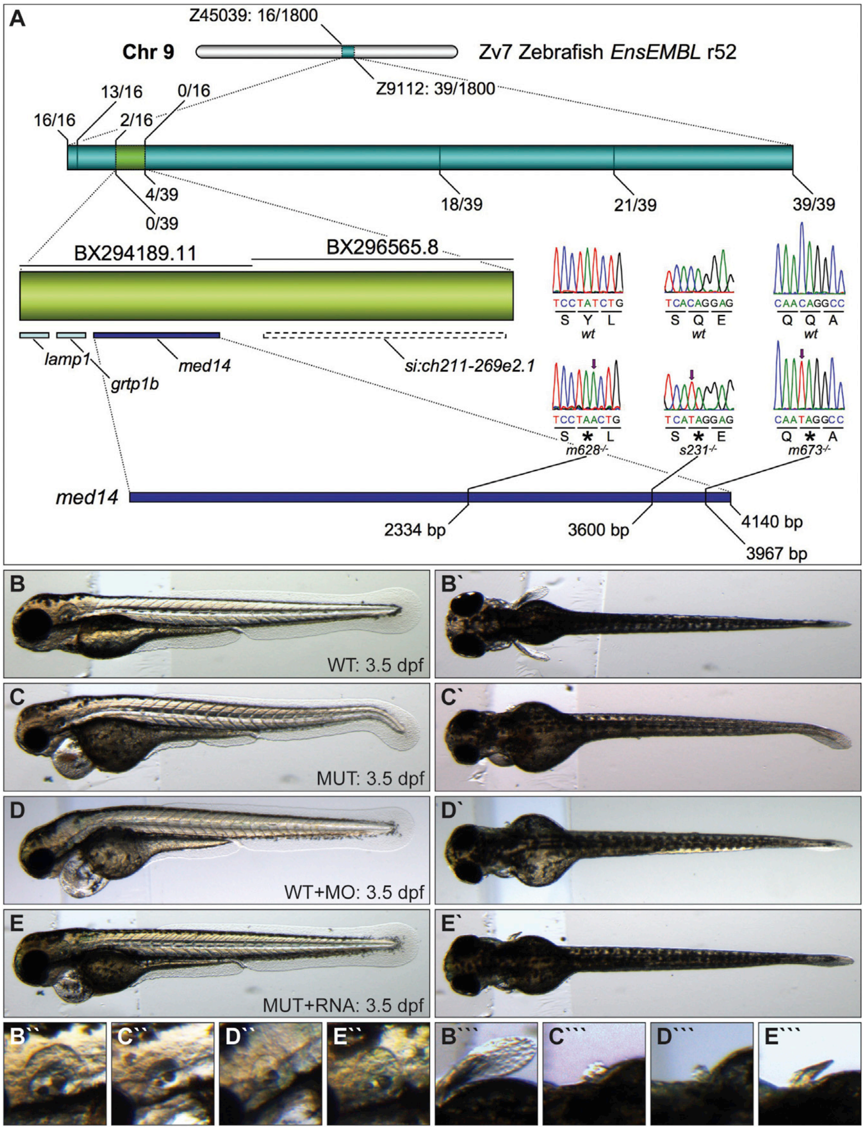

Figure 2. *Logelei* Results from a Mutation in *med14*

(A) Recombination frequency mapping results. Screening of 1,800 map-cross mutant embryos with markers Z45039 and Z9112 revealed 16 and 39 flanking recombinant embryos, respectively. The zero-recombinant region (light green, contained in two bacterial artificial chromosomes [BACs]) was refined with additional markers. Sequencing of *med14* revealed a base pair substitution (arrow) leading to a premature stop codon (*) in each of the three *log* alleles.

(B–E$'''$) Lateral and dorsal views of the otic vesicle and pectoral fin of 3.5 dpf WT (B–B$'''$), *log* mutant (C–C$'''$), *med14* morpholino-injected (D–D$'''$), and *log* mutant injected with *med14* RNA (E–E$'''$) embryos.

vmhc (ventricle) was normal in *log* mutants (Figures 1D–1I). The first observable *log* phenotype, a defect in brain ventricle inflation (Schier et al., 1996), was apparent by 36-hr post-fertilization (hpf). Following this, a developmental delay became apparent in *log* mutants from 48–96 hpf, including absence of pectoral fin elongation and semi-circular canals of the otic vesicle (Figures 1J–1M, arrowheads). Head-trunk angle, a measure of developmental progression (Kimmel et al., 1995), was largely fixed in *log* mutants by 48 hpf (Figure 1N). Despite this arrest in development, there was not an apparent increase in apoptosis or overt proliferative defect (Figure S1).

logelei Is due to Mutation of *med14*

We next sought to determine the causal *log* mutation, which we had previously localized to linkage group 9 (Jin et al., 2007). Further mapping defined a zero recombination region of approximately 100 kb containing four genes. RT-PCR and sequencing from mutant (*s231* and the previously isolated *m628* and *m673* alleles) and wild-type (WT) cDNA pools identified distinct premature stop codons in *med14* in all three alleles (Figure 2A). Injection of *med14* morpholino recapitulated otic vesicle, pectoral fin and cardiac phenotypes, while injection of 300 pg of RNA encoding a WT form of Med14 partially rescued these defects in *log* mutants (Figures 2B–2E$'''$). Further, injection of RNA encoding the *s231*, *m628*, and *m673* forms of *med14* failed to affect appreciable rescue of *log* mutants (results not shown). Taken together, this established that the *log* phenotype is due to mutation of *med14*.

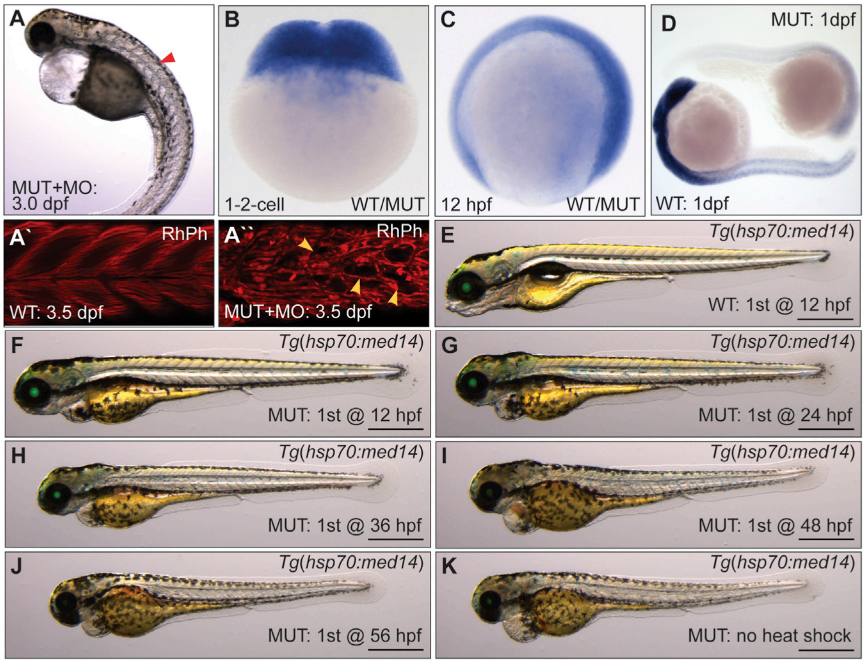

Figure 3. Expression and Timing Requirement of *med14* for Development

(A) Worsening of the *log* mutant phenotype by injection of morpholino (MO) targeting *med14* (red arrowhead denotes somite defect).

(A′ and A″) Somite structure of 3.5 dpf rhodamine phalloidin (RhPh) stained WT and MO-injected mutant embryos. Defects in muscle fiber patterns of mutant embryos injected with MO are shown (yellow arrowheads).

(B–D) RNA ISH analysis shows that *med14* is broadly expressed at the one- and two-cell stage and at 12 hpf (“WT/MUT” denotes unknown genotype). At 24 hpf, broad *med14* expression is undetectable in zygotic *med14* mutants.

(E–K) Temporal rescue of the *log* mutant phenotype using *Tg(hsp70:med14, α-crystallin:EGFP)*, with initial heat shock performed at the specified time, and then every 12 hr following until 5 dpf. Scale bars, 0.5 mm.

Maternal Expression of *med14* Affects Timing of Phenotypic Onset

As we expected that loss of Med14 would have global effects on transcription, we next examined whether *med14* had a maternal function. Injection of *med14* morpholino (which would affect maternal *med14* transcript, but not protein) worsened the *log* mutant phenotype (Figure 3A), notably inducing defects in skeletal muscle fibers (Figures 3A′ and 3A″). *med14* transcript was maternally deposited (Figure 3B) and expressed broadly later in development (Figure 3C; data not shown). ISH analysis in *m628* allele mutants revealed a loss of *med14* expression by 24hpf, suggesting nonsense-mediated decay of mutant transcript and degradation of maternally deposited WT transcript by this time point (Figure 3D). Efforts to deplete WT maternal *med14* transcript by making maternal zygotic mutants through a germline replacement strategy (Ciruna et al., 2002) were not successful (results not shown). To determine whether the *log* mutant phenotype could be alleviated by prolonged expression of *med14*, we generated a transgenic line expressing WT *med14* RNA under control of the inducible *hsp70* promoter, which had no apparent effects on WT development (Figure 3E). In *log* mutants, overexpression of *med14* every 12 hr beginning at 12 hpf until 120 hpf resulted in maximal rescue (Figure 3F). Further analysis revealed that initiation of *med14* overexpression at 24 hpf or later in *log* mutants resulted in progressively more severe phenotypes, with initiation after 48 hpf resulting in no discernable rescue (Figures 3G–3K). These results suggest that maternal Med14 function alleviates the severity of early phenotypes in *log* mutants.

Transcription Is Not Broadly Affected in *log* Mutants

To examine effects on Pol II transcription in *log* mutants, we first assayed expression of *opsin1sw1*, which is initiated in the eye at 2.5 dpf, after developmental defects are readily apparent (Figures 4A and 4B). Although delayed in *log* mutants (data not shown), *opsin1sw1* expression reached a level comparable to WT by 4.0 dpf (Figures 4C and 4D). To test whether transcription could be induced in *log* mutants, we heat shocked *log* mutant *hsp70:EGFP* embryos at 3.5 dpf, after which a robust EGFP fluorescence was observable at 4.0 dpf (Figure 4E). To examine whether cellular transcription and function could continue in the prolonged absence of Med14 function, we carried out transplantation experiments to place transgenic (constitutive *β-actin:EGFP*) *log* mutant cells in WT host embryos, thus circumventing the issue of embryonic lethality. *β-actin:EGFP* expression in *log* mutant cells was evident at 15 dpf (Figure 4F, mutant cells seen in 100% of 50 transplants). Interestingly, cells were found to contribute to structures not present in mutant embryos, such as the semicircular canals. In fish aged up to 2 years, EGFP-+'ve *log* mutant cells persisted, as was evident in many nonpigmented tissues (Figures 4G–4H′). This clearly demonstrated that Med14 was not (at least cell autonomously) required for constitutive transcription or cell survival.

To address in a quantitative manner the extent transcription may be globally affected in *log* mutants, we

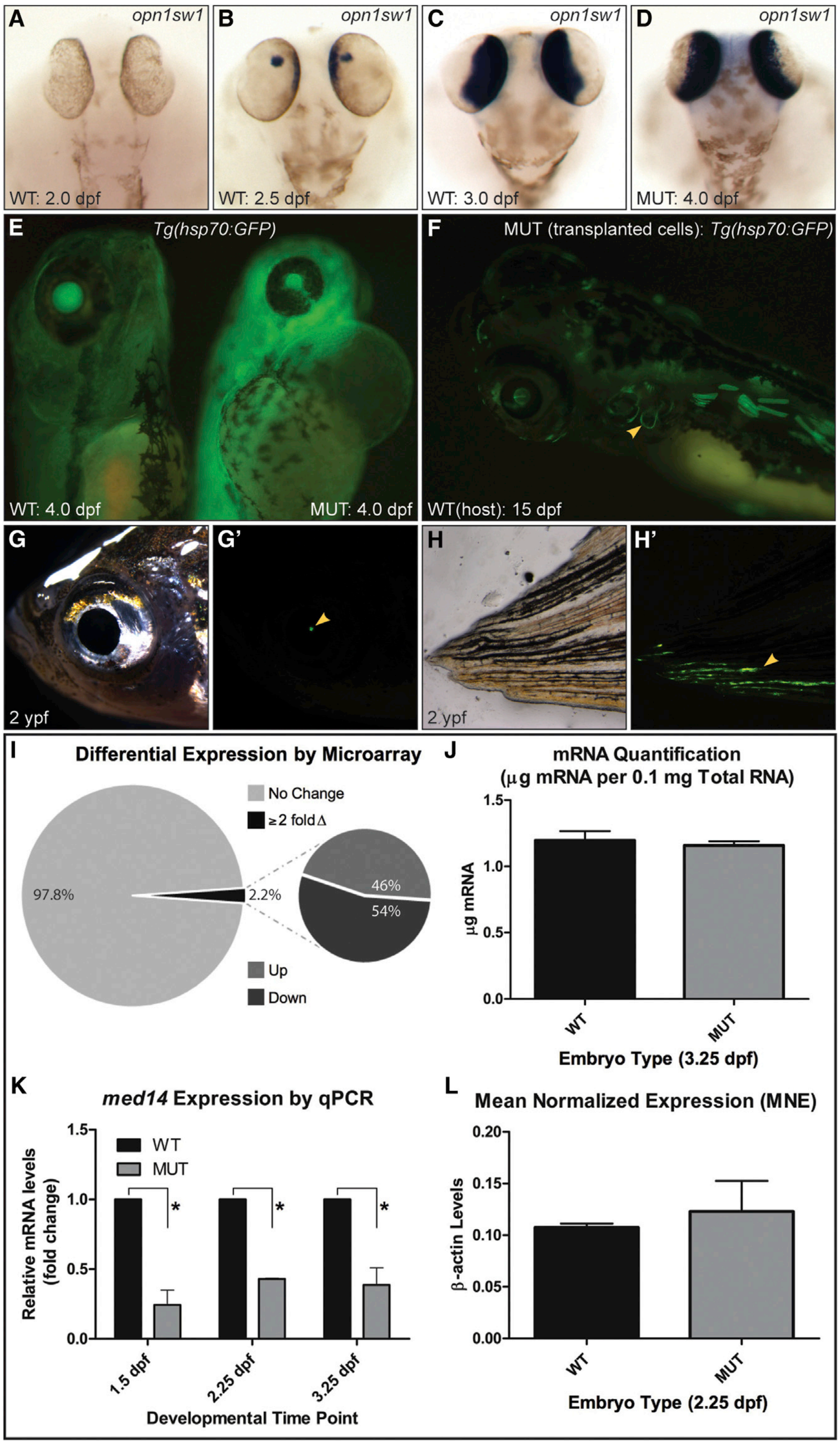

Figure 4. Transcription Is Not Largely Affected in *log* (*med14*) Mutants

(A–D) Expression of *opn1sw1*, initiated by 2.5 dpf in WT embryos, reaches WT levels in *log* mutant embryos by 4.0 dpf.

(E) *Tg*(*hsp70:EGFP*) *log* mutant embryos heat shocked at 3.5 dpf show robust GFP signal.

(F–H′) *log* mutant cells transplanted into WT hosts (traced using a *β-actin:EGFP* transgene) are evident in 15 dpf hosts (including in the semicircular canals, yellow arrowhead in F) and survive until 2 ypf (years post-fertilization, yellow arrowheads).

(I) Summary of results of microarray analysis on cDNA from 2.25 dpf WT versus *log* mutant embryos.

(J) Quantification of mRNA per 0.1mg total RNA at 3.25 dpf from WT and *log* mutant embryos.

(K) Normalized qPCR values for *med14* expression in mutant relative to WT control embryos (*$p < 0.05$ using the one-tailed unpaired Student's t test).

(L) Mean normalized expression (MNE) of *β-actin* in WT and MUT embryos at 2.25 dpf calculated using a universal reference approach. No significant difference ($p = 0.63$) was observed between WT (0.107 ± 0.00380) and *log* MUT (0.123 ± 0.0297) samples ($n = 3$) by one-tailed unpaired Student's t test. For (J)–(L), three biological replicates of 450 (J) or 10 (K and L) embryos were used.

undertook a microarray approach to analyze genome-wide transcript levels at 2.25 dpf (when the *log* mutant phenotype is apparent). Surprisingly, in *log* mutants, only ~2% of genes assayed were differentially expressed (764 of 34,858 with ≥2-fold difference), with a roughly equal number of genes being upregulated or downregulated (Figure 4I). To dismiss the possibility that transcription levels were reduced globally in *log* mutants (Lovén et al., 2012), we assayed mRNA (poly A) levels from an equivalent amounts of total RNA; however, no difference

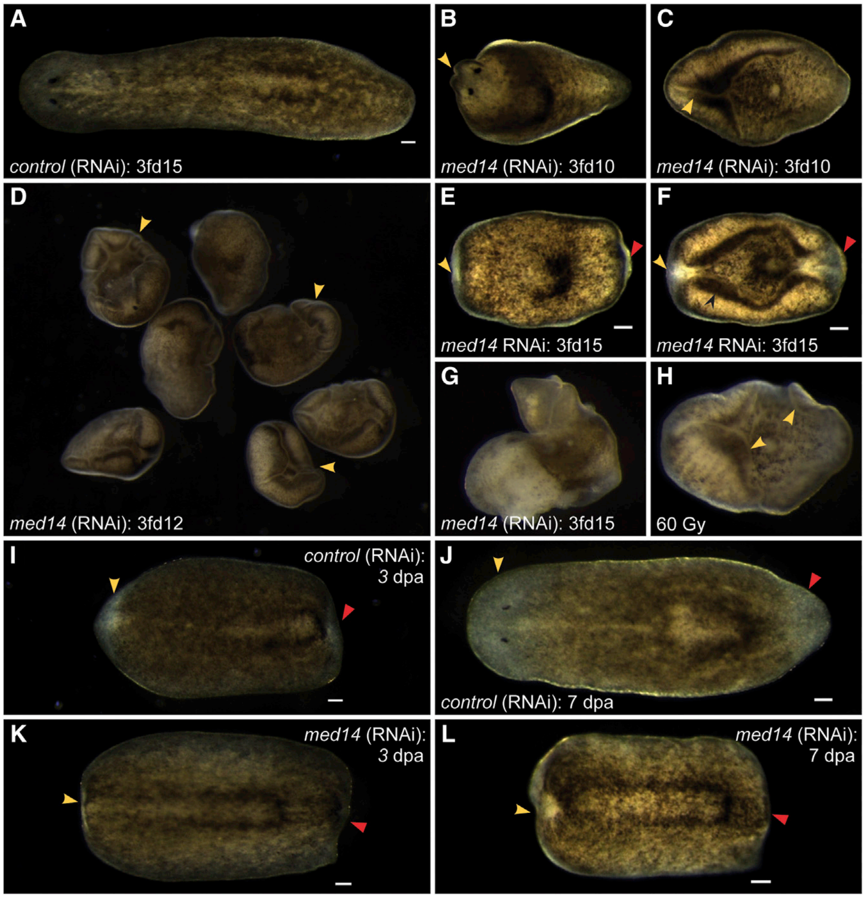

Figure 5. *Smed-med14* Is Required for Tissue Homeostasis and Regeneration in Planarians

(A–F) *med14(RNAi)* animals show defects in homeostasis, with head regression (yellow arrowheads) and ventral curling apparent in 100% of cases by 3fd10.

(G and H) By 3fd12–15, head and tail regression (yellow and red arrowheads) and the beginnings of lysis are apparent in 100% of *med14(RNAi)* animals, similar to what is observed following irradiation-mediated depletion of the stem cell pool.

(I–L) In trunk fragments that are regenerating a head (yellow arrowhead) and a tail (red arrowhead), no regeneration is observed by 7 dpa in *med14(RNAi)* animals. Scale bars, 100 μm.

was evident even at 3.25 dpf (Figure 4J). RNA and DNA spike-ins and genomic PCR to normalize data to total genomic content (cell number) (Bower et al., 2007) was further used to stringently assay mean levels of gene expression. However, global changes in Pol II gene expression were not evident in *med14* mutants (Figure 4L). qRT-PCR analysis validated that from 1.5-3.25 dpf *med14* transcript levels were significantly down-regulated in *log* mutants, in line with our RNA ISH analysis (Figure 4L).

med14 Is Necessary for Stem Cell Maintenance in Planarians

To further decipher Med14 function, we pursued *med14* knockdown in the freshwater planarian *S. mediterranea*. A BLAST search of *S. mediterranea* genome and transcriptomes (Labbé et al., 2012) with both human and zebrafish Med14 sequences revealed a single planarian ortholog, *Smed-med14* (*med14* in this manuscript). When intact planarians were subjected to *med14* RNAi, 100% of animals displayed a ventral curling phenotype by 10 days after the third feeding (3fd10) (Figures 5A–5D). By 3fd15, head and tail regression phenotypes became pronounced in *med14(RNAi)* animals (Figures 5E and 5F), with lysis of the epidermis following (Figure 5G), similar to what is observed following irradiation (Figure 5H). As ventral curling and lysis are hallmarks of a stem cell defect (Reddien et al., 2005), we next examined whether *med14(RNAi)* animals retained regenerative ability, which depends on stem cell function. Following amputation into thirds at 3fd3, regeneration was severely diminished at both 3 and 7 days post-amputation (dpa) in *med14(RNAi)* animals (Figures 5I–5L). As seen in zebrafish embryos, *med14* appeared to be ubiquitously expressed in planarians (Figure 6A). However, dilution of probe and reduction of staining time resulted in a stem cell-like expression pattern (Figure 6A′), in agreement with transcriptome data showing *med14* to be 6.7-fold enriched in stem cells over differentiated tissues (Labbé et al., 2012). Confirmation of *med14* expression in (but not limited to) stem cells was obtained by confocal imaging of the head and tail regions following *med14* fluorescent RNA ISH and PIWI (a marker of stem cells) antibody staining (Figures 6A″ and 6A‴).

To further examine the *med14(RNAi)* phenotype, RNA ISH was carried out for markers of the stem, progeny,

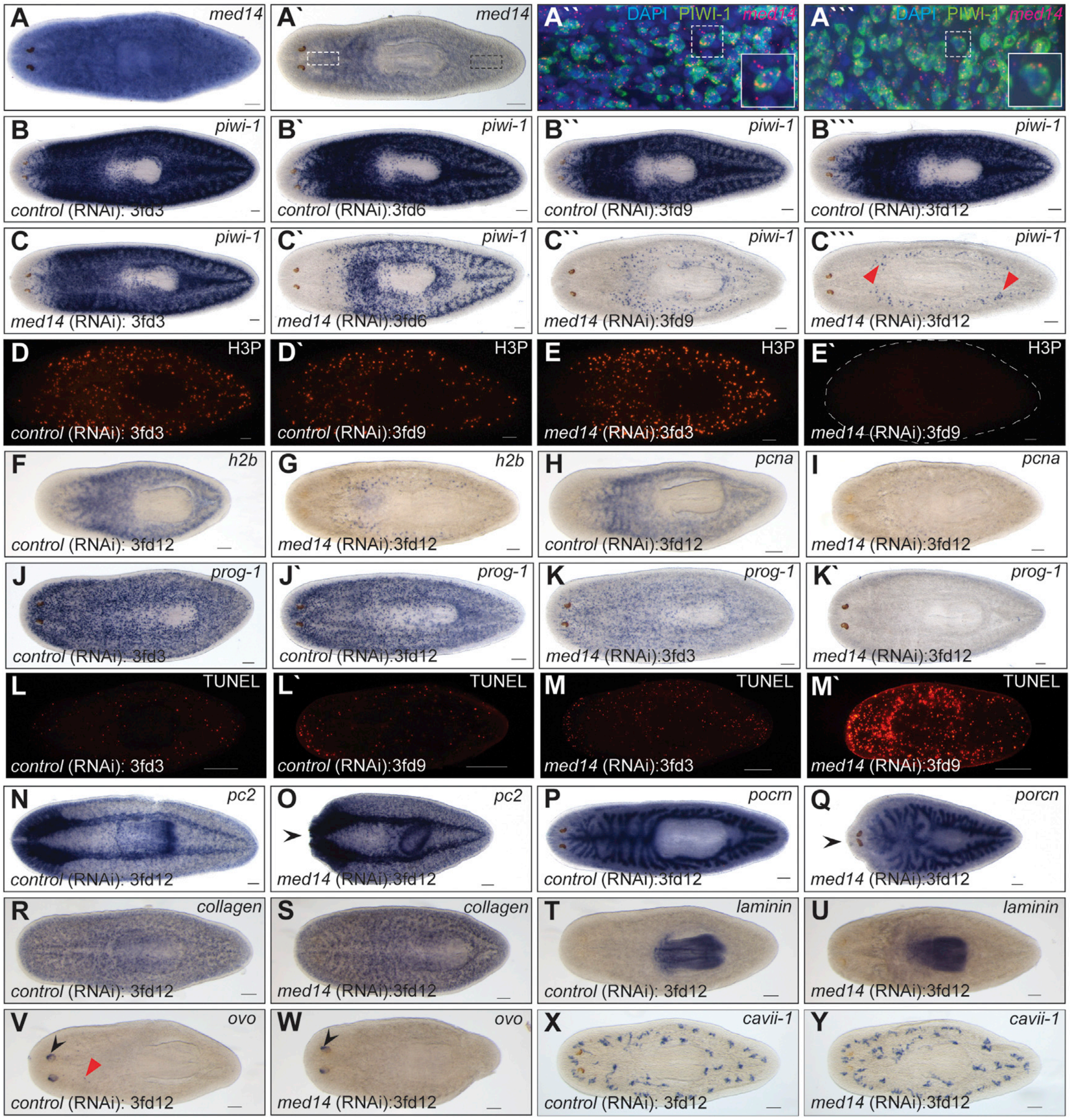

Figure 6. *Smed-med14* Is Necessary for the Maintenance of Adult Stem Cells

(A) ISH for *med14* in wild-type intact animals showing ubiquitous staining.

(A′) A stem cell like expression pattern is evident with reduced staining.

(A″ and A‴) Confocal image at 25× magnification of *med14* fluorescent RNA ISH (red) and PIWI antibody staining (green) in the planarian head (white dashed box in A′) and tail (black dashed box in A′) respectively. The boxed area in each is enlarged for clarification. *med14* is expression in, but not limited to, the stem cell population.

(B–C‴) ISH analysis using a stem cell specific riboprobe (*piwi-1*) during a time course of *med14(RNAi)*. By 3fd12, the stem cell population is largely absent in *med14(RNAi)* animals. The remaining *piwi-1*+ cells at 3fd12 (C‴) may represent primordial germ cells (red arrowheads in M).

(D–E′) Loss of proliferative phosphorylated histone H3 (H3P) +'ve cells in *med14(RNAi)* animals by 3fd9 (E′).

(F–I) Expression of S-phase markers *h2b* and *pcna* in WT and *med14* RNAi animals at 3fd12.

(J–K′) By 3fd3, the progenitor cell population in *med14(RNAi)* animals (marked by *prog-1* expression) is reduced compared with controls and completely absent by 3fd12.

(L–M′) Increased cell death by 3fd9 as observed by whole-mount TUNEL analysis in *med14(RNAi)* animals.

(legend continued on next page)

and somatic compartments of the animal. Analysis of the stem cell marker *piwi-1* (Reddien et al., 2005) revealed a reduction in *piwi-1*-expressing cells in *med14(RNAi)* worms by 3fd6 and a complete absence by 3fd12 (Figures 6B–6C‴). However, expression of *piwi-1* could still be detected in two dorsal-lateral regions that may represent primordial germ cells (Wang et al., 2010) (Figure 6C‴, red arrowheads), suggesting that Med14 is not required directly for *piwi-1* expression. As the stem cell compartment in planaria is the only one that undergoes proliferation (Reddien and Sánchez Alvarado, 2004), expression of markers of mitosis (phosphorylated histone H3-H3P) and S-phase (*h2b* and *pcna*) was assayed. As expected for a complete stem cell loss, proliferation was not evident in *med14(RNAi)* animals by 3fd9/12 (Figures 6D–6I). The loss of *piwi-1*-expressing cells following *med14* RNAi may have been as a consequence of differentiation of the stem cell compartment. To examine this, we analyzed the expression of *prog-1*, which marks the immediate daughters of stem cells (Eisenhoffer et al., 2008), in control and *med14(RNAi)* worms (Figures 6J–6K′). A reduction to complete absence in the *prog-1*-positive progenitor cell population that mirrored observations of the *piwi-1* stem cell population was found, suggesting that the stem and progenitor cell pools were rapidly depleted following loss of *med14*. In support of this model, widespread apoptosis was evident in *med14(RNAi)* animals by 3fd9 (Figures 6L–6M′). In contrast, knockdown of *med14* function had no effect on differentiated cells as exemplified by expression of CNS (*pc2*), gut (*porcn*), muscle (*collagen*), pharynx (*laminin*), eye (*ovo*), and protonephridia (*cavii-1*) markers at 3fd12, with the general patterns of expression and organ shapes being normal (Figures 6N–6Y). These results suggest that loss of *med14* has a specific effect on the stem cell population and that transcription in general is not compromised.

To investigate a more general requirement for Mediator in stem cell maintenance, we performed an RNAi knockdown screen of 11 additional Mediator subunits, allowing analysis of 12 of the 25 conserved proteins (Figure S2 and table within). A ventral curling phenotype was observed for five subunits tested: *med7*, *med12*, *med17*, *med19*, and *med27* (Figures S2A–S2F and table within). In all cases, depletion of the *piwi-1*-positive stem cell population was evident by 3fd12 (Figures S2A–S3F′). No phenotypes were observed following knockdown of the remaining six subunits (data not shown). When considered collectively, a stem cell phenotype was apparent following knockdown of a subunit from each Mediator domain.

Med14 Is Required for Maintenance of Progenitor Populations in Zebrafish

We next re-examined the zebrafish *log* mutant phenotype, with a focus on stem/progenitor cells and regeneration. Using RNA ISH, we first assayed expression of the retinal stem cell marker *mz98* (Cerveny et al., 2010) and found that while present at 2.25 dpf, expression was lost by 3.25 dpf in *log* mutants (Figures 7A–7D). Analysis of the hematopoietic stem cell marker *cmyb* (Bertrand et al., 2008) in the ventral trunk/tail region of the embryo revealed a loss of expression in *log* mutants by 3.25 dpf (Figures 7E–7H). Similarly, the putative gut stem cell marker *lgr4* (Hirose et al., 2011) showed a severe reduction in expression in *log* mutants at 3.25 dpf (Figures 7I and 7J). To assess a possible role for Med14 in vertebrate regeneration, we employed amputation of the zebrafish embryonic tailfin (Kawakami et al., 2004). Following resection of the tailfin at 2 dpf, we observed no appreciable regrowth of *log* mutant fins by 4 dpf (Figures 7K and 7L).

We next assessed readouts of stem cell function. As indicated by o-Dianisidine staining, the pronounced expansion of the red blood cell population evident in WT embryos from 48–96 hpf did not occur in *med14* mutants (Figures 7M–7R). We next examined growth of the zebrafish ventricle from 24–48 hpf, which has recently been shown to include addition of cells from second heart field progenitors (Lazic and Scott, 2011). Cardiomyocyte cell number was significantly reduced in 60 hpf *med14* morphant hearts (Figures 7S–7U). To test whether the expression of WT *med14* in cardiomyocytes could rescue the *log* heart defect, we generated a cardiomyocyte-specific *cmlc2:med14* transgenic line. Despite forced expression of WT *med14* RNA in mutant hearts well before the onset of phenotypic defects, no rescue was observed (Figure 7V). Consistent with these results, expression of the second heart field progenitor marker *ltbp3* (Zhou et al., 2011) was greatly reduced in *log* mutant embryos at 48 hpf (Figures 7W and 7X), suggesting that *med14* is required in heart progenitor cells (prior to *cmlc2* expression).

(N–Y) Normal expression of markers of differentiated cell types in *med14(RNAi)* animals as evident for the nervous system (*pc2*), gut (*porcn*), muscle (*collagen*), pharynx (*laminin*), eyes (*ovo*), and protonephridia (*cavii-1*). Head regression is evident in some treated worms (black arrow heads in O and Q). Eye progenitors (red arrow head in V) are not observed in *med14(RNAi)* animals despite *ovo* expression in the eye spots (black arrow heads in V and W).
Scale bars, 100 μm. See also Figure S2.

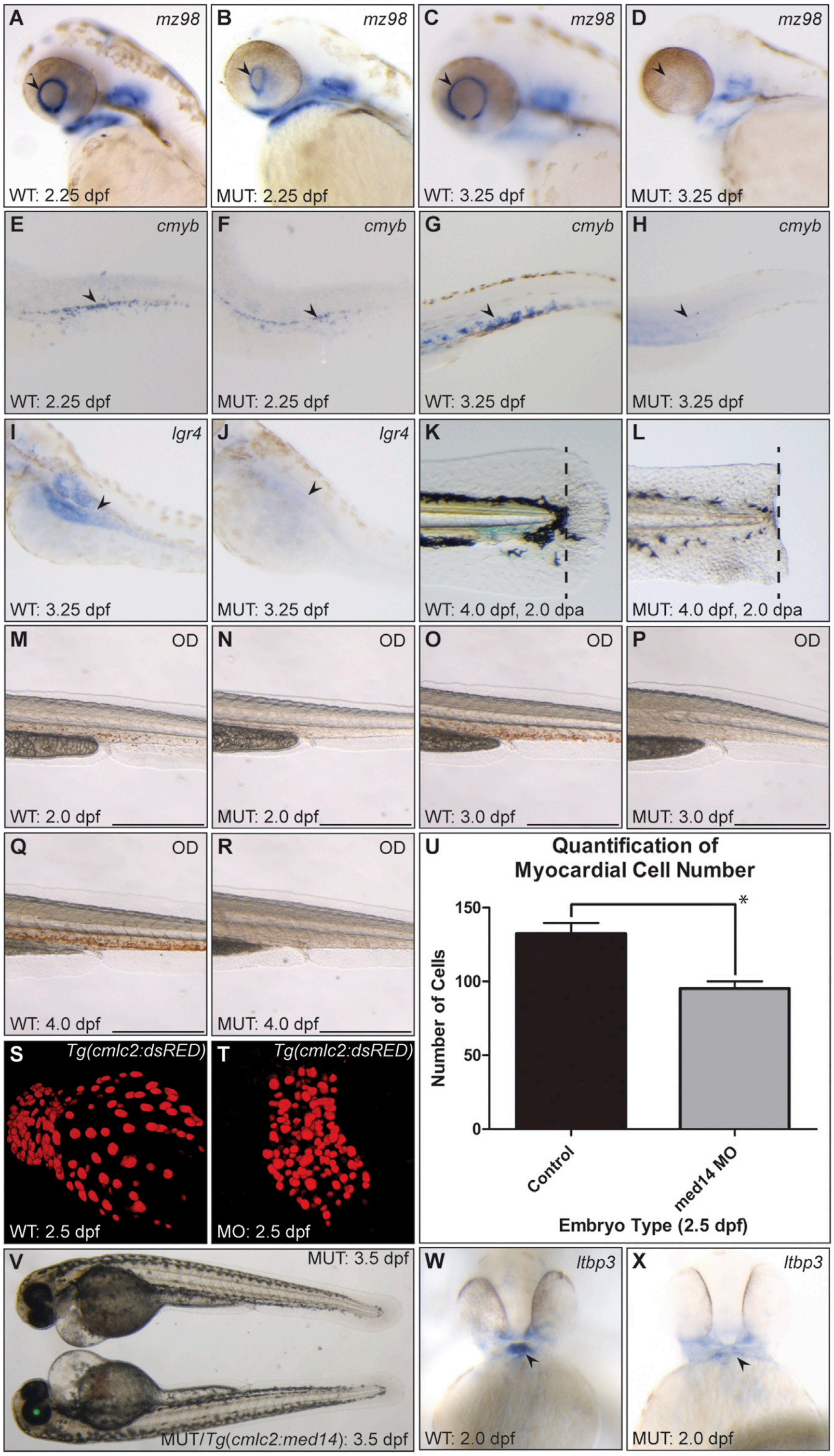

Figure 7. A Conserved Requirement for *med14* in the Maintenance of Stem Cell Populations

(A–D) Expression of retinal stem cell marker *mz98* (arrowhead) is present, but reduced in *log* mutants at 2.25 dpf, and absent at 3.25 dpf.

(E–H) Expression of the hematopoietic stem cell marker *cmyb* is initiated in 2.25 dpf *log* mutant embryos (arrowhead), but largely absent by 3.25 dpf.

(I and J) Expression of the putative gut stem cell marker *lgr4* is not observed in 3.25 dpf *log* mutant embryos (arrowhead, note expression in WT).

(K and L) Robust tail fin regeneration in WT as compared with *log* mutant embryos at 4 dpf following amputation (at area of dotted line) at 2 dpf.

(M–R) o-Dianisidane staining of red blood cells in trunks of WT and *log* mutant embryos. Scale bars, 0.5 mm.

(S and T) Confocal projections of 2.5 dpf *Tg(cmcl2:nlsDsRedExpress)* WT and *log* morphant hearts.

(U) Quantification of myocardial cell number in WT and morphant hearts shows a significant decrease in morphants ($p = 0.0017$, $n = 6$ for both conditions).

(V) Cardiac edema and heart defects remain in 3.5 dpf *Tg (cmlc2:med14, α-crystallin:EGFP) log* mutant embryos.

(W and X) Expression of the second heart field marker *ltbp3* in the arterial pole of the heart (arrowhead) is reduced in *log* mutants at 48 hpf.

DISCUSSION

In this study, we have shown in two animal models (including one adult one) that the Mediator complex plays a key role in stem cell maintenance. To our knowledge, our planarian work is the first broad in vivo survey of Mediator function in stem cells. Further, our results represent a whole-organism view that helps address controversies in Mediator function in general transcription versus having more specialized functions.

Our results, we believe, provide the first description of the consequences of the complete loss of function of *med14* in metazoa. A zebrafish *med14* mutant was reported as having relatively minor defects in eye development (Dürr et al., 2006). However, this mutant (*hi2143*) resulted from an intronic retroviral insertion, and online pictures available show roughly normal development (http://web.mit.edu/hopkins/group6.html). As this is a much weaker phenotype than seen in *log* mutants, the insertion allele is likely hypomorphic. A mouse *med14* mutant made from a gene trap ESC line reported no overt phenotypes at day 10.5 (Cox et al., 2010). However, our analysis of this gene trap line by RT-PCR revealed that WT *Med14* transcript was still present, indicating that this allele was not a null (data not shown). In contrast, RNAi-mediated knockdown of *rgr1* (*med14*) in *C. elegans* has been reported to mimic loss of Pol II, which contrasts our findings in zebrafish and planarians (Shim et al., 2002). Of course, it remains possible that the *med14* alleles used in our studies are not true nulls. All three encode C-terminally truncated versions of Med14, with the *m673* and *s231* alleles resembling the original (viable) yeast *rgr1* (*med14*) mutant. This truncation prevented association of Rgr1 with many Mediator Tail components, resulting in a largely "Tail-less" Mediator (Li et al., 1995; Sakai et al., 1990). In contrast, a yeast *rgr1* null (deletion) allele results in lethality (Sakai et al., 1990). As the *m628* allele creates a large truncation of Med14 and further results in pronounced loss of transcript, we are confident that this results in a full loss of function. Recent analysis of Mediator structure has shown that Med14 is a key interface that contacts all three Mediator modules (Tsai et al., 2014), with Med14 being essential for activity of a biochemically reconstituted Mediator complex (Cevher et al., 2014). As we were unable to examine the full (maternal zygotic) zebrafish *med14* mutant phenotype, the observed onset of *log* phenotypes is likely due to when depletion of maternal protein brings Med14 levels below a threshold level in certain cell types. As discussed below, this may be especially relevant to genes with "superenhancers," which may require a higher level of Mediator in a cell.

Our work suggests that stem and progenitor cells may be especially sensitive to Mediator complex function and that the *log* phenotype is due to loss of stem cells. This will require more extensive study, including cell fate and lineage tracing approaches. Interestingly, our transplantation experiments clearly show that cells lacking Med14 can survive to adulthood and contribute to many tissues. Gene expression analysis in *log* mutants did not reveal effects on stem-cell specific genes; however, analysis of whole embryo transcriptomes is not well suited to detect changes in small populations of cells. While the nature of the stem cell defect in zebrafish *med14* mutants requires further clarification, the effects of Med14 loss on planarian adult stem cell function is more evident. This is associated with a loss of proliferation and increased apoptosis in animals, but not an increase in *prog-1* expression. Our results therefore suggest a model where *piwi-1*-positive stem cells are depleted following *med14* RNAi, perhaps via cell death. This requires further investigation, as does the cellular autonomy (in stem cells versus a niche) of Med14 function.

The role of Mediator in general versus specialized aspects of Pol II-based transcription has been the subject of debate. Work in yeast suggests that core components of Mediator are required for most Pol II-mediated transcription and that Mediator subunits are localized upstream of most promoters (Andrau et al., 2006; Holstege et al., 1998). In contrast, Mediator has also been shown to be localized to a very limited fraction (perhaps 3%) of promoter elements (Fan and Struhl, 2009), regulating expression of a low percentage of genes (Young et al., 2009). Similar to our microarray results, expression of only a few hundred genes is misregulated in yeast *rgr1* (*med14*) CTD deletion mutants (Young et al., 2009). However, it is also true that not all Mediator subunits are equal: loss of "core" subunits such as Med17 may have more severe effects than loss of other subunits (Holstege et al., 1998).

In cultured ESCs and induced pluripotent stem cells, multiple Mediator (and cohesin) complex subunits have been shown to play essential roles in stem cell maintenance (Kagey et al., 2010). In vivo, Med1, Med14, Med21, and Cdk8 have been shown to be required in hair follicle stem cells, plant meristem, mouse blastocysts, and tumor cells, respectively (Adler et al., 2012; Autran et al., 2002; Nakajima et al., 2013; Tudor et al., 1999). Our planarian results demonstrate a requirement for Mediator subunits from all structural components (Head, Middle, Tail, and Kinase) in adult stem cells. Specific roles for other "core" components of the transcriptional machinery in stem cells have been described. Alterations in expression of the TBP-associated factors (TAFs) have been associated with pluripotency and skeletal muscle differentiation, and expression of many Mediator subunits is decreased during myotube differentiation (Deato et al., 2008; Maston et al., 2012).

The global expression of Mediator subunit (and TAF) genes in vivo has not been carefully examined to date. It is possible that distinct Mediator complexes, which contain different combinations of subunits, may have differential function in stem cells versus other contexts.

How may Mediator ultimately affect stem cell maintenance? The ESC genome has been described as existing in a poised state, with enhancers of many developmental genes having a bivalent chromatin status (Bernstein et al., 2006; Rada-Iglesias et al., 2011). This may be essential for the coordinated maintenance and differentiation of stem cells in response to the appropriate cues (Lagha et al., 2013). It is interesting to note that a variant form of the Pol II complex containing Gdown1 (Pol II(G)) requires Mediator for activator-based Pol II transcription (Hu et al., 2006; Jishage et al., 2012). Interestingly, Pol II(G) is highly enriched at genes with poised Pol II. The Polycomb/PRC2 complex has been shown to inhibit the bivalent poised status of genes in ESCs (Jia et al., 2012). PRC2 and cohesin complexes can physically interact (Strübbe et al., 2011) and may competitively bind DNA (Cunningham et al., 2012). A function of Mediator/cohesin may be to prevent association of PRC2 activity with pluripotency-associated genes. Finally, the recent description of stem cell-enriched super-enhancers is of special interest. These atypically large enhancer regions are highly enriched for Mediator (Med1) occupancy, with Mediator being essential for their organization (Lovén et al., 2013; Whyte et al., 2013). Enrichment for Mediator occupancy at *Nanog* enhancers that form complex interactions with multiple sites in the genome is similarly associated with pluripotency (Apostolou et al., 2013). It is therefore tempting to speculate that Mediator is intimately involved with establishing the epigenetic landscape essential for pluripotency and stem cell maintenance. In this context, super-enhancer-regulated genes, which contain high levels of Mediator, may be especially sensitive to Mediator levels and/or complex organization.

EXPERIMENTAL PROCEDURES

Mutant and Transgenic Zebrafish Lines

Zebrafish were housed and handled as per Canadian Council on Animal Care and Hospital for Sick Children Laboratory Animal Services guidelines. The *s231* allele of *med14* (Jin et al., 2007), *hsp70:EGFP* (Halloran et al., 2000), and *Ola.Actb:Hsa.HRAS-EGFP*^{vu119} (Cooper et al., 2005) lines have been previously described. *m628* and *m673* were acquired from the Zebrafish International Resource Center (ZIRC). We generated *hsp70:med14, cryaa:EGFP*^{hsc10}, and *myl7:med14, cryaa:EGFP* transgenics using standard Tol2 transgenesis (Kawakami, 2005). Full-length zebrafish *med14* coding sequence was subcloned downstream of a *hsp70* (Halloran et al., 2000) or *myl7/cmlc2* (Huang et al., 2003) promoter between minimal Tol2 transposon arms (Urasaki et al., 2006) in a pBluescript backbone vector carrying a *cryaa:EGFP* cassette (Kurita et al., 2003). Heat shock was performed for 30 min in 37°C media. *Tg(myl7:nlsDsRedExpress*^{hsc4}*)* embryos were used to quantify cardiomyocyte number as previously described (Takeuchi et al., 2011).

Positional Cloning of *log*

Mutant embryos collected from incrosses of heterozygous breeding pairs from a *log*^{s231}/WIK mapcross were screened for recombination at two flanking markers (Z45039 and Z9112). Additional simple sequence length polymorphism (SSLP) markers were designed based on genomic dinucleotide repeats. Recombinant mutant embryos were screened with these markers to further narrow the genomic region. Coding sequence of candidate genes within the region were cloned by RT-PCR and sequenced to uncover mutations in *s231*, *m628*, and *m673* alleles.

Morpholino and mRNA Microinjections, Transplantation

For rescue/overexpression analysis, full-length WT or mutant (*s231*, *m628*, *m673*) *med14* coding sequence was subcloned into pCS2+ vector for in vitro transcription using the mMESSAGE mMACHINE kit (Applied Biosystems) and injected at 300 pg per embryo. A *med14* morpholino targeting the ATG translational start site (5′-CCGAACCGATCTGAACTGGAGCCAT-3′) was purchased from Gene Tools, with 6 ng injected per embryo. For transplantation experiments, donor embryos from a *log*^{m628}+/−; *Ola.Actb:Hsa.HRAS-EGFP*^{vu119} +/− cross were used, with cells transplanted into multiple regions of WT host embryos at 4 hpf. Donor embryos were kept paired with corresponding host embryos to identify *log* mutant donors that were EGFP+'ve.

RNA ISH

RNA ISH using DIG-labeled antisense RNA probes was performed as previously described (Pearson et al., 2009; Thisse and Thisse, 2008). Fluorescent ISH (FISH) in planarians using the alkaline phosphatase (AP) substrate Fast Blue was performed as previously described (Cowles et al., 2013). Probe fragments used are described in Supplemental Information.

Microarray Analysis of Gene Expression

Two-color microarray experiments were performed by the UHN Microarray Facility using the Zebrafish (v.3) 44 k Gene Expression Microarray Platform (Agilent). cDNA was generated from total RNA isolated from pools of 20 WT or *m628* mutant embryos at 54 hpf, with two biological replicates used. Microarray results were analyzed using Genespring v.11.0.1 (Agilent), with data normalized using Agilent's Spatial Detrending and Lowess normalization. After normalization and averaging, data were filtered such that only probes that were between the 20th and 100th percentile of the distribution of intensities in both samples for either group were kept. Statistical significance for differential expression between sample groups was set at $p < 0.05$.

Quantitative Real-Time PCR and RNA Quantification

Reverse transcription reactions were conducted on total RNA extracted from 10 embryos using a SuperScript III Reverse Transcriptase Kit (Invitrogen). Quantitative real-time PCR was performed in triplicate using an Applied Biosystems Real-time PCR system (Life Technologies) with Platinum SYBR Green PCR Master Mix (Invitrogen). For relative quantification of *med14* (SE: CAGAGACTGTGTTCGCATCA, AS: TCAGACAGAACTGCACATT CC), the comparative C_T method was used (Schmittgen and Livak, 2008). Primer pairs for ubiquitously expressed *β-actin* were used as a reference (Tang et al., 2007). Methods for analysis of mean normalized expression (Bower et al., 2007) are described in the Supplemental Information. For mRNA quantification, total RNA was extracted from 3.25 dpf WT and *log* mutant embryos using Trizol. Poly A mRNA was then isolated from 0.1 mg of total RNA using a QIAGEN Oligotex mRNA mini kit (QIAGEN), followed by quantification using a Nano-Drop spectrophotometer (Thermo Scientific).

Apoptosis, Proliferation, and Cell Cycle Assays

Apoptotic cells were detected in zebrafish embryos using an In Situ Cell Death Detection Kit-AP (Roche). For cell proliferation experiments, embryos were incubated on ice for 10 min in 10-mM bromodeoxyuridine (BrdU) (Sigma) in 15% DMSO. Mouse anti-BrdU (BD Biosciences) primary and Alexa Fluor 568 anti-mouse secondary (Invitrogen) antibodies were used. Whole-mount terminal deoxynucleotidyl transferase dUTP nick end labeling (TUNEL) staining was carried out in planarians as previously described (Pellettieri et al., 2010).

Flow Cytometry Analysis

Single-cell suspensions were fixed in ethanol and stained with propidium iodide (0.1 mg/ml in Hank's balanced salt solution [HBSS] with 0.6% NP40 and 2mg/ml RNase A) for 30 min at room temperature. A FACSLSRII flow cytometer (BD Biosciences) was used, with data analyzed using FlowJo software (Tree Star). Three samples were run for both conditions (wild-type and mutant) at each time point (54 and 78 hpf).

Immunohistochemistry

A 1:100 dilution of rhodamine phalloidin (Life Technologies) was used in zebrafish. Immunostaining with anti-phosphohistone H3 (H3ser10p, 1:500 dilution; Millipore) with a 1:200 dilution of a goat anti-rabbit IgG HRP secondary antibody (Jackson ImmunoResearch) to label mitotic cells in planarians and monoclonal mouse anti-PIWI (1:1000, gift from Jochen Rink) with a 1:300 dilution of a goat anti-mouse HRP secondary antibody (Jackson ImmunoResearch) to label stem cells was performed as previously reported (Newmark and Sánchez Alvarado, 2000). For detection of the presence of hemoglobin, PFA-fixed zebrafish embryos were stained in the dark for 10 min in PBS containing o-Dianisidine dihydrochloride (0.6 mg/ml; Sigma), sodium acetate (0.01 M, pH 4.5), H_2O_2 (0.65%), and ethanol (40%).

Planarian RNAi and Homeostasis/Regeneration Assays

The asexual clonal line CIW4 of *S. mediterranea* was maintained as previously described (Sanchez Alvarado and Newmark, 1998). For production of dsRNA for RNAi experiments, a pRT4P vector containing either full-length (*med14*) or partial (remaining Mediator subunits) cDNA was expressed in a HT115 bacterial strain as previously reported (Sánchez Alvarado and Newmark, 1999). RNAi food was made by mixing a pellet of dsRNA-expressing bacteria from 30 ml of culture (OD600 of 0.8) with 300 μl of 70% liver paste. For both homeostasis and regeneration assays, RNAi food was fed to worms every 3 days for three feedings. Animals were amputated into three equal pieces 3 days after the last feeding (3fd3). For zebrafish regeneration assays, fin primordia of anesthetized embryos were cut immediately posterior to the notochord at 48 hpf using a number 17 (square ended) scalpel blade as previously described (Kawakami et al., 2004).

ACCESSION NUMBERS

MIAME-compliant microarray data have been submitted to Gene Expression Omnibus under accession number GSE58042.

SUPPLEMENTAL INFORMATION

Supplemental Information includes Supplemental Experimental Procedures and two figures and can be found with this article online at http://dx.doi.org/10.1016/j.stemcr.2015.02.006.

AUTHOR CONTRIBUTIONS

J.T.A.B., B.J.P., and I.C.S. conceived and designed the experiments. J.T.A.B. performed the experiments. J.T.A.B., B.J.P., and I.C.S. wrote the paper.

ACKNOWLEDGMENTS

We thank members of the Scott, Pearson, and Ciruna labs for helpful suggestions and Angela Morley for zebrafish husbandry. Shu Jun Zhu and David Brown provided expert help with planarian experiments. Brian Ciruna provided guidance for germline replacement studies, and Xin Lou aided in cardiomyocyte counts. Carl Vitanen (UHN Microarray Facility) aided in analysis of microarray data, and Sherry Zhao (SickKids-UHN Flow Cytometry Facility) helped with fluorescence-activated cell sorting. We thank Brian Ciruna for providing *Ola.Actb:Hsa.HRAS-EGFP* zebrafish and the ZIRC for providing *m628* and *m673* alleles. Anti-SMEDWI-1 antibody was a kind gift of Jochen Rink. This work was supported by grant funding from the Ontario Institute for Cancer Research (to B.J.P., number IA-026) and the Natural Sciences and Engineering Research Council of Canada (to I.C.S., RGPIN 341545).

Received: May 30, 2014
Revised: February 10, 2015
Accepted: February 11, 2015
Published: March 12, 2015

REFERENCES

Adler, A.S., McCleland, M.L., Truong, T., Lau, S., Modrusan, Z., Soukup, T.M., Roose-Girma, M., Blackwood, E.M., and Firestein,

R. (2012). CDK8 maintains tumor dedifferentiation and embryonic stem cell pluripotency. Cancer Res. *72*, 2129–2139.

Andrau, J.C., van de Pasch, L., Lijnzaad, P., Bijma, T., Koerkamp, M.G., van de Peppel, J., Werner, M., and Holstege, F.C. (2006). Genome-wide location of the coactivator mediator: Binding without activation and transient Cdk8 interaction on DNA. Mol. Cell *22*, 179–192.

Apostolou, E., Ferrari, F., Walsh, R.M., Bar-Nur, O., Stadtfeld, M., Cheloufi, S., Stuart, H.T., Polo, J.M., Ohsumi, T.K., Borowsky, M.L., et al. (2013). Genome-wide chromatin interactions of the Nanog locus in pluripotency, differentiation, and reprogramming. Cell Stem Cell *12*, 699–712.

Autran, D., Jonak, C., Belcram, K., Beemster, G.T., Kronenberger, J., Grandjean, O., Inzé, D., and Traas, J. (2002). Cell numbers and leaf development in Arabidopsis: a functional analysis of the STRUWWELPETER gene. EMBO J. *21*, 6036–6049.

Bernstein, B.E., Mikkelsen, T.S., Xie, X., Kamal, M., Huebert, D.J., Cuff, J., Fry, B., Meissner, A., Wernig, M., Plath, K., et al. (2006). A bivalent chromatin structure marks key developmental genes in embryonic stem cells. Cell *125*, 315–326.

Bertrand, J.Y., Kim, A.D., Teng, S., and Traver, D. (2008). CD41+ cmyb+ precursors colonize the zebrafish pronephros by a novel migration route to initiate adult hematopoiesis. Development *135*, 1853–1862.

Bower, N.I., Moser, R.J., Hill, J.R., and Lehnert, S.A. (2007). Universal reference method for real-time PCR gene expression analysis of preimplantation embryos. Biotechniques *42*, 199–206.

Cerveny, K.L., Cavodeassi, F., Turner, K.J., de Jong-Curtain, T.A., Heath, J.K., and Wilson, S.W. (2010). The zebrafish flotte lotte mutant reveals that the local retinal environment promotes the differentiation of proliferating precursors emerging from their stem cell niche. Development *137*, 2107–2115.

Cevher, M.A., Shi, Y., Li, D., Chait, B.T., Malik, S., and Roeder, R.G. (2014). Reconstitution of active human core Mediator complex reveals a critical role of the MED14 subunit. Nat. Struct. Mol. Biol. *21*, 1028–1034.

Ciruna, B., Weidinger, G., Knaut, H., Thisse, B., Thisse, C., Raz, E., and Schier, A.F. (2002). Production of maternal-zygotic mutant zebrafish by germ-line replacement. Proc. Natl. Acad. Sci. USA *99*, 14919–14924.

Cooper, M.S., Szeto, D.P., Sommers-Herivel, G., Topczewski, J., Solnica-Krezel, L., Kang, H.C., Johnson, I., and Kimelman, D. (2005). Visualizing morphogenesis in transgenic zebrafish embryos using BODIPY TR methyl ester dye as a vital counterstain for GFP. Dev. Dyn. *232*, 359–368.

Cowles, M.W., Brown, D.D., Nisperos, S.V., Stanley, B.N., Pearson, B.J., and Zayas, R.M. (2013). Genome-wide analysis of the bHLH gene family in planarians identifies factors required for adult neurogenesis and neuronal regeneration. Development *140*, 4691–4702.

Cox, B.J., Vollmer, M., Tamplin, O., Lu, M., Biechele, S., Gertsenstein, M., van Campenhout, C., Floss, T., Kühn, R., Wurst, W., et al. (2010). Phenotypic annotation of the mouse X chromosome. Genome Res. *20*, 1154–1164.

Cunningham, M.D., Gause, M., Cheng, Y., Noyes, A., Dorsett, D., Kennison, J.A., and Kassis, J.A. (2012). Wapl antagonizes cohesin binding and promotes Polycomb-group silencing in Drosophila. Development *139*, 4172–4179.

Deato, M.D., Marr, M.T., Sottero, T., Inouye, C., Hu, P., and Tjian, R. (2008). MyoD targets TAF3/TRF3 to activate myogenin transcription. Mol. Cell *32*, 96–105.

Dürr, K., Holzschuh, J., Filippi, A., Ettl, A.K., Ryu, S., Shepherd, I.T., and Driever, W. (2006). Differential roles of transcriptional mediator complex subunits Crsp34/Med27, Crsp150/Med14 and Trap100/Med24 during zebrafish retinal development. Genetics *174*, 693–705.

Eisenhoffer, G.T., Kang, H., and Sánchez Alvarado, A. (2008). Molecular analysis of stem cells and their descendants during cell turnover and regeneration in the planarian Schmidtea mediterranea. Cell Stem Cell *3*, 327–339.

Fan, X., and Struhl, K. (2009). Where does mediator bind in vivo? PLoS ONE *4*, e5029.

Halloran, M.C., Sato-Maeda, M., Warren, J.T., Su, F., Lele, Z., Krone, P.H., Kuwada, J.Y., and Shoji, W. (2000). Laser-induced gene expression in specific cells of transgenic zebrafish. Development *127*, 1953–1960.

Hengartner, C.J., Thompson, C.M., Zhang, J., Chao, D.M., Liao, S.M., Koleske, A.J., Okamura, S., and Young, R.A. (1995). Association of an activator with an RNA polymerase II holoenzyme. Genes Dev. *9*, 897–910.

Hentges, K.E. (2011). Mediator complex proteins are required for diverse developmental processes. Semin. Cell Dev. Biol. *22*, 769–775.

Hirose, K., Shimoda, N., and Kikuchi, Y. (2011). Expression patterns of lgr4 and lgr6 during zebrafish development. Gene Expr. Patterns *11*, 378–383.

Ho, R.K., and Kimmel, C.B. (1993). Commitment of cell fate in the early zebrafish embryo. Science *261*, 109–111.

Holstege, F.C., Jennings, E.G., Wyrick, J.J., Lee, T.I., Hengartner, C.J., Green, M.R., Golub, T.R., Lander, E.S., and Young, R.A. (1998). Dissecting the regulatory circuitry of a eukaryotic genome. Cell *95*, 717–728.

Hu, X., Malik, S., Negroiu, C.C., Hubbard, K., Velalar, C.N., Hampton, B., Grosu, D., Catalano, J., Roeder, R.G., and Gnatt, A. (2006). A Mediator-responsive form of metazoan RNA polymerase II. Proc. Natl. Acad. Sci. USA *103*, 9506–9511.

Huang, C.J., Tu, C.T., Hsiao, C.D., Hsieh, F.J., and Tsai, H.J. (2003). Germ-line transmission of a myocardium-specific GFP transgene reveals critical regulatory elements in the cardiac myosin light chain 2 promoter of zebrafish. Dev. Dyn. *228*, 30–40.

Jia, J., Zheng, X., Hu, G., Cui, K., Zhang, J., Zhang, A., Jiang, H., Lu, B., Yates, J., 3rd, Liu, C., et al. (2012). Regulation of pluripotency and self- renewal of ESCs through epigenetic-threshold modulation and mRNA pruning. Cell *151*, 576–589.

Jin, S.W., Herzog, W., Santoro, M.M., Mitchell, T.S., Frantsve, J., Jungblut, B., Beis, D., Scott, I.C., D'Amico, L.A., Ober, E.A., et al. (2007). A transgene-assisted genetic screen identifies essential regulators of vascular development in vertebrate embryos. Dev. Biol. *307*, 29–42.

Jishage, M., Malik, S., Wagner, U., Uberheide, B., Ishihama, Y., Hu, X., Chait, B.T., Gnatt, A., Ren, B., and Roeder, R.G. (2012). Transcriptional regulation by Pol II(G) involving mediator and competitive interactions of Gdown1 and TFIIF with Pol II. Mol. Cell *45*, 51–63.

Kagey, M.H., Newman, J.J., Bilodeau, S., Zhan, Y., Orlando, D.A., van Berkum, N.L., Ebmeier, C.C., Goossens, J., Rahl, P.B., Levine, S.S., et al. (2010). Mediator and cohesin connect gene expression and chromatin architecture. Nature *467*, 430–435.

Kawakami, K. (2005). Transposon tools and methods in zebrafish. Dev. Dyn. *234*, 244–254.

Kawakami, A., Fukazawa, T., and Takeda, H. (2004). Early fin primordia of zebrafish larvae regenerate by a similar growth control mechanism with adult regeneration. Dev. Dyn. *231*, 693–699.

Kelleher, R.J., 3rd, Flanagan, P.M., and Kornberg, R.D. (1990). A novel mediator between activator proteins and the RNA polymerase II transcription apparatus. Cell *61*, 1209–1215.

Kim, Y.J., Björklund, S., Li, Y., Sayre, M.H., and Kornberg, R.D. (1994). A multiprotein mediator of transcriptional activation and its interaction with the C-terminal repeat domain of RNA polymerase II. Cell *77*, 599–608.

Kimmel, C.B., Ballard, W.W., Kimmel, S.R., Ullmann, B., and Schilling, T.F. (1995). Stages of embryonic development of the zebrafish. Dev. Dyn. *203*, 253–310.

Kurita, R., Sagara, H., Aoki, Y., Link, B.A., Arai, K., and Watanabe, S. (2003). Suppression of lens growth by alphaA-crystallin promoter-driven expression of diphtheria toxin results in disruption of retinal cell organization in zebrafish. Dev. Biol. *255*, 113–127.

Labbé, R.M., Irimia, M., Currie, K.W., Lin, A., Zhu, S.J., Brown, D.D., Ross, E.J., Voisin, V., Bader, G.D., Blencowe, B.J., and Pearson, B.J. (2012). A comparative transcriptomic analysis reveals conserved features of stem cell pluripotency in planarians and mammals. Stem Cells *30*, 1734–1745.

Lagha, M., Bothma, J.P., Esposito, E., Ng, S., Stefanik, L., Tsui, C., Johnston, J., Chen, K., Gilmour, D.S., Zeitlinger, J., and Levine, M.S. (2013). Paused Pol II coordinates tissue morphogenesis in the Drosophila embryo. Cell *153*, 976–987.

Lazic, S., and Scott, I.C. (2011). Mef2cb regulates late myocardial cell addition from a second heart field-like population of progenitors in zebrafish. Dev. Biol. *354*, 123–133.

Li, Y., Bjorklund, S., Jiang, Y.W., Kim, Y.J., Lane, W.S., Stillman, D.J., and Kornberg, R.D. (1995). Yeast global transcriptional regulators Sin4 and Rgr1 are components of mediator complex/RNA polymerase II holoenzyme. Proc. Natl. Acad. Sci. USA *92*, 10864–10868.

Lovén, J., Orlando, D.A., Sigova, A.A., Lin, C.Y., Rahl, P.B., Burge, C.B., Levens, D.L., Lee, T.I., and Young, R.A. (2012). Revisiting global gene expression analysis. Cell *151*, 476–482.

Lovén, J., Hoke, H.A., Lin, C.Y., Lau, A., Orlando, D.A., Vakoc, C.R., Bradner, J.E., Lee, T.I., and Young, R.A. (2013). Selective inhibition of tumor oncogenes by disruption of super-enhancers. Cell *153*, 320–334.

Maston, G.A., Zhu, L.J., Chamberlain, L., Lin, L., Fang, M., and Green, M.R. (2012). Non-canonical TAF complexes regulate active promoters in human embryonic stem cells. elife *1*, e00068.

Nakajima, T., Inui, S., Fushimi, T., Noguchi, F., Kitagawa, Y., Reddy, J.K., and Itami, S. (2013). Roles of MED1 in quiescence of hair follicle stem cells and maintenance of normal hair cycling. J. Invest. Dermatol. *133*, 354–360.

Newmark, P.A., and Sánchez Alvarado, A. (2000). Bromodeoxyuridine specifically labels the regenerative stem cells of planarians. Dev. Biol. *220*, 142–153.

Nichols, J., Zevnik, B., Anastassiadis, K., Niwa, H., Klewe-Nebenius, D., Chambers, I., Schöler, H., and Smith, A. (1998). Formation of pluripotent stem cells in the mammalian embryo depends on the POU transcription factor Oct4. Cell *95*, 379–391.

Parameswaran, M., and Tam, P.P. (1995). Regionalisation of cell fate and morphogenetic movement of the mesoderm during mouse gastrulation. Dev. Genet. *17*, 16–28.

Pearson, B.J., Eisenhoffer, G.T., Gurley, K.A., Rink, J.C., Miller, D.E., and Sánchez Alvarado, A. (2009). Formaldehyde-based whole-mount in situ hybridization method for planarians. Dev. Dyn. *238*, 443–450.

Pellettieri, J., Fitzgerald, P., Watanabe, S., Mancuso, J., Green, D.R., and Sánchez Alvarado, A. (2010). Cell death and tissue remodeling in planarian regeneration. Dev. Biol. *338*, 76–85.

Rada-Iglesias, A., Bajpai, R., Swigut, T., Brugmann, S.A., Flynn, R.A., and Wysocka, J. (2011). A unique chromatin signature uncovers early developmental enhancers in humans. Nature *470*, 279–283.

Reddien, P.W., and Sánchez Alvarado, A. (2004). Fundamentals of planarian regeneration. Annu. Rev. Cell Dev. Biol. *20*, 725–757.

Reddien, P.W., Oviedo, N.J., Jennings, J.R., Jenkin, J.C., and Sánchez Alvarado, A. (2005). SMEDWI-2 is a PIWI-like protein that regulates planarian stem cells. Science *310*, 1327–1330.

Sakai, A., Shimizu, Y., Kondou, S., Chibazakura, T., and Hishinuma, F. (1990). Structure and molecular analysis of RGR1, a gene required for glucose repression of Saccharomyces cerevisiae. Mol. Cell. Biol. *10*, 4130–4138.

Sanchez Alvarado, A., and Newmark, P.A. (1998). The use of planarians to dissect the molecular basis of metazoan regeneration. Wound repair and regeneration *6*, 413–420.

Sánchez Alvarado, A., and Newmark, P.A. (1999). Double-stranded RNA specifically disrupts gene expression during planarian regeneration. Proc. Natl. Acad. Sci. USA *96*, 5049–5054.

Schier, A.F., Neuhauss, S.C., Harvey, M., Malicki, J., Solnica-Krezel, L., Stainier, D.Y., Zwartkruis, F., Abdelilah, S., Stemple, D.L., Rangini, Z., et al. (1996). Mutations affecting the development of the embryonic zebrafish brain. Development *123*, 165–178.

Schmittgen, T.D., and Livak, K.J. (2008). Analyzing real-time PCR data by the comparative C(T) method. Nat. Protoc. *3*, 1101–1108.

Shim, E.Y., Walker, A.K., and Blackwell, T.K. (2002). Broad requirement for the mediator subunit RGR-1 for transcription in the Caenorhabditis elegans embryo. J. Biol. Chem. *277*, 30413–30416.

Strübbe, G., Popp, C., Schmidt, A., Pauli, A., Ringrose, L., Beisel, C., and Paro, R. (2011). Polycomb purification by in vivo biotinylation tagging reveals cohesin and Trithorax group proteins as interaction partners. Proc. Natl. Acad. Sci. USA *108*, 5572–5577.

Takahashi, K., and Yamanaka, S. (2006). Induction of pluripotent stem cells from mouse embryonic and adult fibroblast cultures by defined factors. Cell *126*, 663–676.

Takeuchi, J.K., Lou, X., Alexander, J.M., Sugizaki, H., Delgado-Olguín, P., Holloway, A.K., Mori, A.D., Wylie, J.N., Munson, C., Zhu, Y., et al. (2011). Chromatin remodelling complex dosage modulates transcription factor function in heart development. Nat. Commun. *2*, 187.

Tang, R., Dodd, A., Lai, D., McNabb, W.C., and Love, D.R. (2007). Validation of zebrafish (Danio rerio) reference genes for quantitative real-time RT-PCR normalization. Acta Biochim. Biophys. Sin. (Shanghai) *39*, 384–390.

Thisse, C., and Thisse, B. (2008). High-resolution in situ hybridization to whole-mount zebrafish embryos. Nat. Protoc. *3*, 59–69.

Thompson, C.M., and Young, R.A. (1995). General requirement for RNA polymerase II holoenzymes in vivo. Proc. Natl. Acad. Sci. USA *92*, 4587–4590.

Thompson, C.M., Koleske, A.J., Chao, D.M., and Young, R.A. (1993). A multisubunit complex associated with the RNA polymerase II CTD and TATA-binding protein in yeast. Cell *73*, 1361–1375.

Tsai, K.L., Tomomori-Sato, C., Sato, S., Conaway, R.C., Conaway, J.W., and Asturias, F.J. (2014). Subunit architecture and functional modular rearrangements of the transcriptional mediator complex. Cell *157*, 1430–1444.

Tudor, M., Murray, P.J., Onufryk, C., Jaenisch, R., and Young, R.A. (1999). Ubiquitous expression and embryonic requirement for RNA polymerase II coactivator subunit Srb7 in mice. Genes Dev. *13*, 2365–2368.

Tutter, A.V., Kowalski, M.P., Baltus, G.A., Iourgenko, V., Labow, M., Li, E., and Kadam, S. (2009). Role for Med12 in regulation of Nanog and Nanog target genes. J. Biol. Chem. *284*, 3709–3718.

Urasaki, A., Morvan, G., and Kawakami, K. (2006). Functional dissection of the Tol2 transposable element identified the minimal cis-sequence and a highly repetitive sequence in the subterminal region essential for transposition. Genetics *174*, 639–649.

Wang, Y., Stary, J.M., Wilhelm, J.E., and Newmark, P.A. (2010). A functional genomic screen in planarians identifies novel regulators of germ cell development. Genes Dev. *24*, 2081–2092.

Whyte, W.A., Orlando, D.A., Hnisz, D., Abraham, B.J., Lin, C.Y., Kagey, M.H., Rahl, P.B., Lee, T.I., and Young, R.A. (2013). Master transcription factors and mediator establish super-enhancers at key cell identity genes. Cell *153*, 307–319.

Young, E.T., Yen, K., Dombek, K.M., Law, G.L., Chang, E., and Arms, E. (2009). Snf1-independent, glucose-resistant transcription of Adr1-dependent genes in a mediator mutant of Saccharomyces cerevisiae. Mol. Microbiol. *74*, 364–383.

Zhou, Y., Cashman, T.J., Nevis, K.R., Obregon, P., Carney, S.A., Liu, Y., Gu, A., Mosimann, C., Sondalle, S., Peterson, R.E., et al. (2011). Latent TGF-β binding protein 3 identifies a second heart field in zebrafish. Nature *474*, 645–648.

Stem Cell Reports

Supplemental Information

An In Vivo Requirement for the Mediator Subunit Med14 in the Maintenance of Stem Cell Populations

Jeffrey T.A. Burrows, Bret J. Pearson, and Ian C. Scott

Supplemental Figure 1

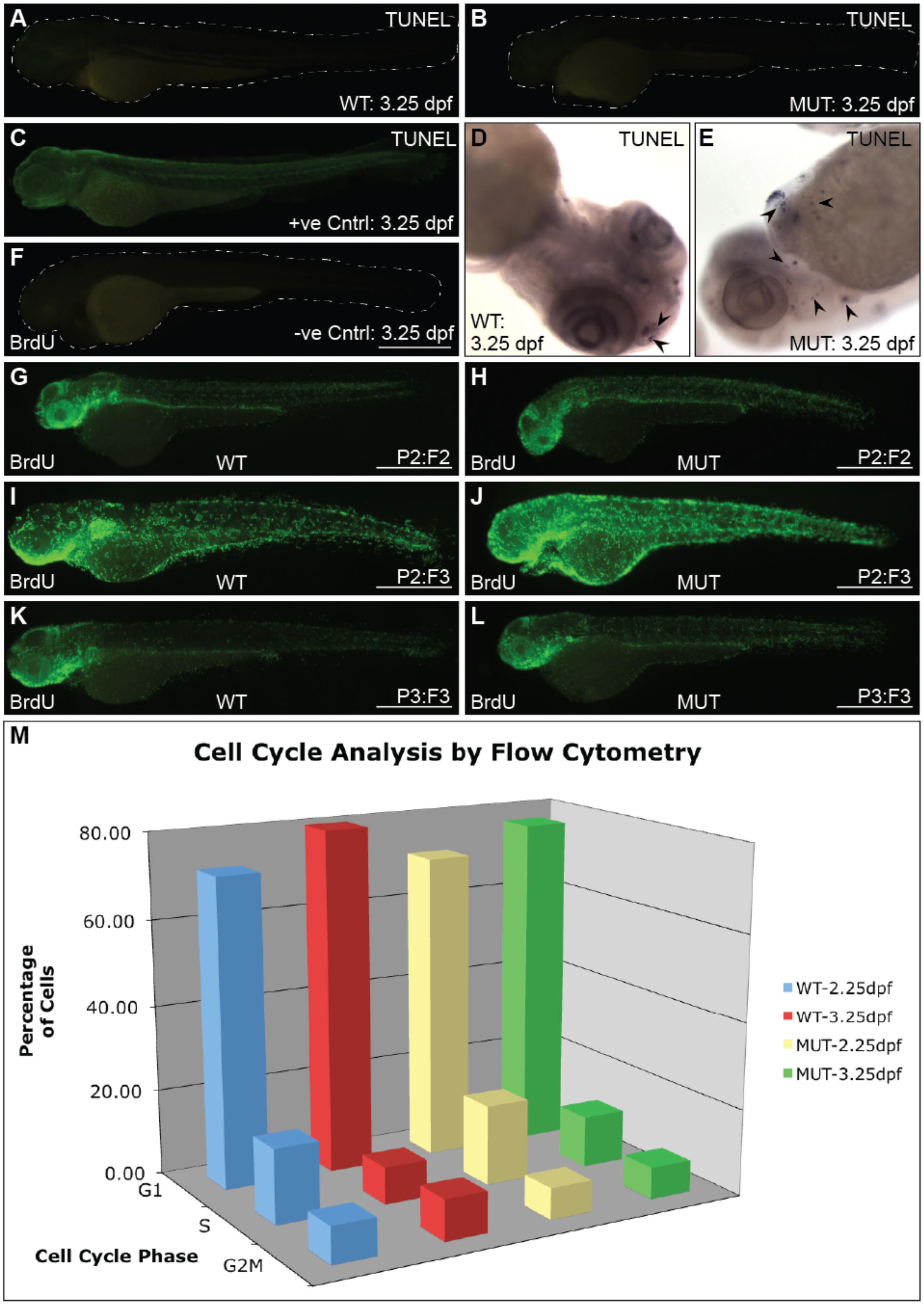

Supplemental Figure 1 (related to Figure 1): Cell Death, Cell Proliferation and Cell Cycle Parameters are Largely Normal in *log* Mutants.

(A-E) TUNEL assay for cell death in 3.25dpf embryos (embryos outlined in A and B). No TUNEL-positive cells were observed in wildtype or mutant embryos. Robust labeling of cell nuclei was evident in the positive control (DNase treated 3.25 wildtype embryos, C). (D-E) AP (Alkaline Phosphatase) amplification of signal revealed some TUNEL-positive cells in 3.25dpf wildtype and mutant embryos (black arrowheads). (F-L) Embryos were pulsed with BrdU for 10 minutes at 2dpf and fixed either immediately (P2F2) or after a 24-hour chase period (P2F3). A BrdU pulse and subsequent fix at 3dpf was also carried out (P3F3). (M) Results of cell cycle analysis by flow cytometry for DNA content on dissociated wildtype and mutant embryos. Percentage values are as follows (mean +/- std error for G1, S, G2M respectively): 2.25dpf WT (blue bars) (71.95 +/- 2.77, 17.28 +/- 2.74, 8.4 +/- 0.75), 3.25dpf WT (red bars) (79.98 +/- 2.05, 8.59 +/- 0.97, 9.42 +/- 0.36), 2.25dpf MUT (yellow bars) (71.28 +/- 0.78, 18.58 +/- 0.56, 7.20 +/- 0.30), 3.25 MUT (green bars) (77.05 +/- 1.54, 11.84 +/- 1.40, 7.31 +/- 0.30). Note that no significant difference is observed between comparisons between matched cell cycle phases for wildtype and mutant at 2.25dpf ($p=0.82, 0.66, 0.17$ for WT G1 vs. MUT G1, WT S vs. MUT S and WT G2M vs. MUT G2M respectively) and that changes in percentage of cells in each stage of the cell cycle follow the same trend in wildtype and mutant embryos when going from 2.25 to 3.25dpf ($n = 3$ biological replicates, 50 embryos per sample). Scale bars in (F-L) represent 0.5 mm.

Supplemental Figure 2

SUBUNIT	*med8*	*med17*	*med19*	*med1*	*med7*	*med31*	*med14*	*med23*	*med27*	*med12*	*CDK8*	*Cyclin C*
DOMAIN	Head	Head	Head	Middle	Middle	Middle	Tail	Tail	Tail	Kinase	Kinase	Kinase
SC DEFECT	No	Yes	Yes	No	Yes	No	Yes	No	Yes	Yes	No	No

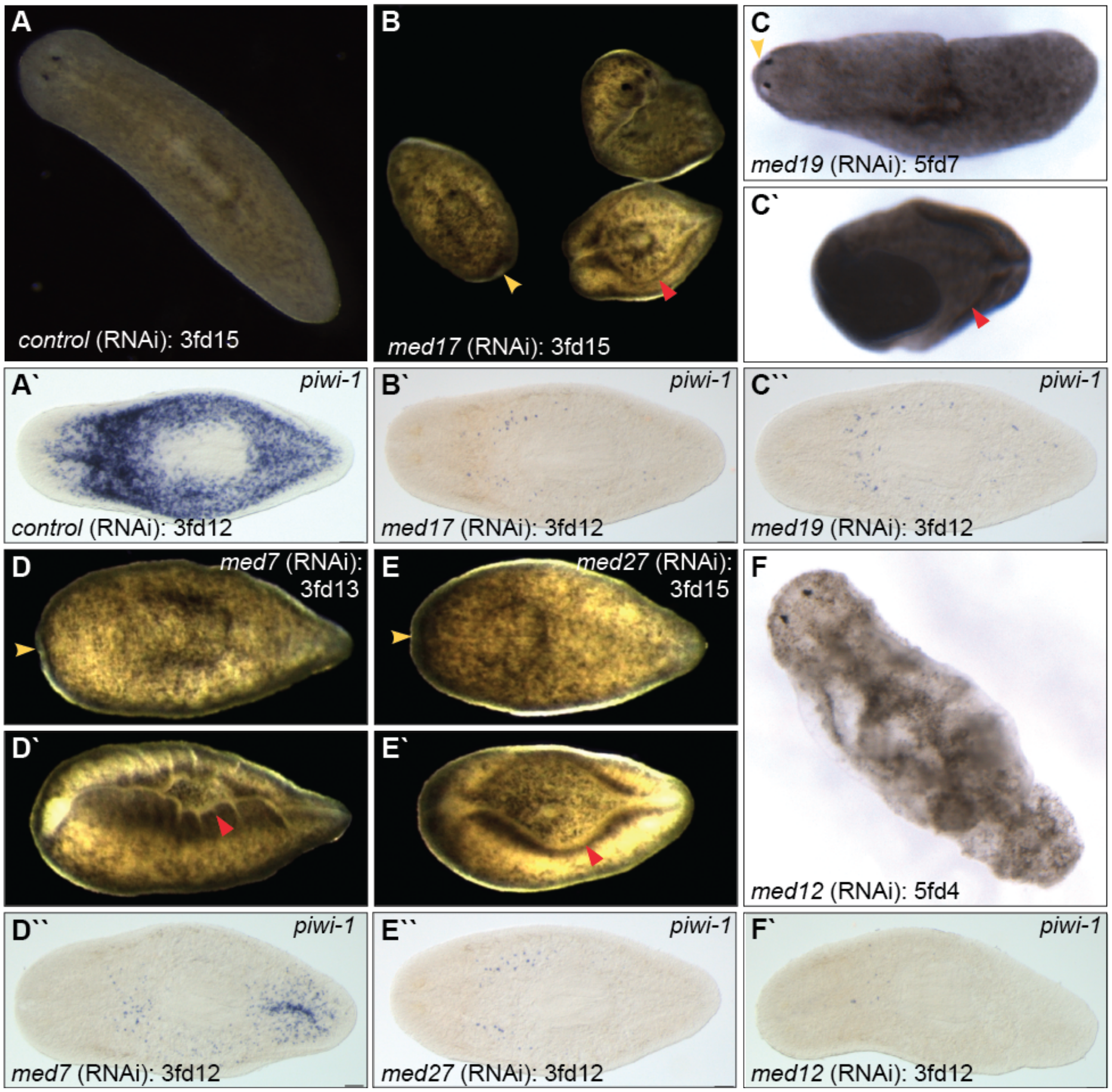

Supplemental Figure 2 (related to Figure 6): Multiple Mediator Components in Planarians are Required for Stem Cell Maintenance.

Table - A stem cell (SC) defect was observed for highlighted subunits. (A-F`) Results of a screen aimed to determine whether the requirement for stem cell maintenance is

specific to *med14* or a general feature of Mediator Complex. (A, A`) Representative live and *piwi-1* ISH image of a control RNAi animal, respectively. (B, B`) Representative *med17* (RNAi) planarian at displaying a stem cell defect phenotype. Note head regression (yellow arrowhead) and ventral curling (red arrowhead, B) as well as loss of *piwi-1* expression (B`). Dorsal and ventral views (C, C`) respectively of an early stem cell defect phenotype observed in representative *med19* (RNAi) animal. (C``) ISH for *piwi-1* confirms loss of the stem cell population in *med19* (RNAi) planaria. (D, D`) Dorsal and ventral views respectively of a representative *med7* (RNAi) animal displaying a stem cell defect phenotype. (D``) A varying degree of *piwi-1* expression loss was observed by ISH in *med7* (RNAi) planaria ranging from sporadic patches to complete absence. (E, E`) Dorsal and ventral views, respectively, of a representative *med27* (RNAi) animal. (E``) ISH for *piwi-1* confirms loss of the stem cell population following *med27* RNAi. (F) Live dorsal view of a representative *med12* (RNAi) planarian displaying a lysis phenotype. (F`) *piwi-1* ISH image confirms loss of the stem cell population in a representative *med12* (RNAi) animal.

SUPPLEMENTAL EXPERIMENTAL PROCEDURES

Probes for RNA *in situ* Hybridization

Templates for zebrafish *med14* (ZDB-GENE-030131-8867), *opn1sw1* (ZDB-GENE-991109-25), *mz98* (*col15a1b,* ZDB-GENE- 070912-689), *cmyb* (ZDB-GENE- 991110-14), *lgr4* (ENSDARG00000060542) and *ltbp3* (ZDB-GENE-060526-130) riboprobes were generated by RT-PCR from 72hpf cDNA template using gene-specific primers and subcloned into pGEM-T Easy vector (Promega). Previously described riboprobe templates for *myl7* (*cmlc2*, ZDB-GENE-991019-3), *vmhc* (ZDB-GENE-991123-5) and *myh6* (*amhc*, ZDB-GENE-031112-1) were used (Yelon et al., 1999). Probes were made via *in vitro* transcription from linearized DNA templates using a DIG RNA Labeling Kit (Roche). The planarian *med14* homolog was found using BLAST against the assembled *S. mediterranea* genome (Robb et al., 2008). It was then cloned by RT-PCR from cDNA using gene-specific primers into pRT4P vector. The remaining planarian Mediator subunits (*med8*, *med17*, *med19*, *med1*, *med7*, *med31*, *med23*, *med27*, *med12*, *Cyclin C* and *CDK8*) were cloned in same manner. Riboprobe was generated by PCR amplification and reverse transcription using a DIG labeling mix. Additional planarian riboprobes used have been previously characterized (*smedwi-1/piwi-1* (Reddien et al., 2005), *prog-1* (Eisenhoffer et al., 2008) *pc2* and porcn (Gurley et al., 2008), *laminin* (Cebria and Newmark, 2007), *collagen* (Witchley et al., 2013), *cavii-1* (Rink et al., 2011), *ovo* (Lapan and Reddien, 2012), *pcna* (Orii et al., 2005) and *h2b* (Guo et al., 2006)).

Mean Normalized Expression Analysis

Mean Normalized Expression (MNE = $[E_{GFP\text{-}RNA}^{(GFP\text{-}RNA\ C_T)} / E_{\beta\text{-}actin}^{(\beta\text{-}actin\ C_T)}] / GCN$) for *β-actin* was calculated using a universal reference approach which employs 2 exogenous spikes as pseudo-housekeeping genes for normalization of the gene expression (Bower et al., 2007). Briefly, GFP-RNA (10ng) and DNA (1.0ng) spikes were added to homogenized embryos prior to simultaneous extraction of RNA and DNA using a Qiagen AllPrep DNA/RNA Mini Kit (Qiagen). RNA samples were treated with DNase as part of the extraction protocol (RNase-Free DNase Set, Qiagen). Genome Copy Number (GCN $= E_{GFP\text{-}DNA}^{(GFP\text{-}DNA\ C_T)} / E_{NC\text{-}DNA}^{(NC\text{-}DNA\ C_T)}$) was calculated from qPCR measurement of genomic copies of a conserved non-coding region (NC-DNA, SE: TCTCCGGACTCCACAACTT, AS: AACAGCTCGAGTGGATTGTG) (Shin et al., 2005) normalized to the GFP-DNA spike (SE: TGTTATGGTGTTCAATGCTTCTC, AS: TGACTTCAGCACGTGTCTTGT). Following RT-PCR of the RNA fractions, the expression of *β-actin* (using the same primers as previous stated) and the GFP-RNA (using the same primers for the GFP-DNA spike) were measured by qPCR and then adjusted by GCN. In all cases amplification Efficiency (E) was assessed through a dilution series. The DNA spike was generated through PCR amplification of the GFP coding sequence contained in a pCS2+ plasmid (SE: GTCAGTGGAGAGGGTGAAGG, AS: AAAGGGCAGATTGTGTGGAC). The RNA spike was generated off the same plasmid by *in vitro* transcription as described for the *med14* overexpression/rescue analysis.

SUPPLEMENTAL REFERENCES

Bower, N.I., Moser, R.J., Hill, J.R., and Lehnert, S.A. (2007). Universal reference method for real-time PCR gene expression analysis of preimplantation embryos. Biotechniques *42*, 199-206.

Cebria, F., and Newmark, P.A. (2007). Morphogenesis defects are associated with abnormal nervous system regeneration following roboA RNAi in planarians. Development *134*, 833-837.

Eisenhoffer, G.T., Kang, H., and Sanchez Alvarado, A. (2008). Molecular analysis of stem cells and their descendants during cell turnover and regeneration in the planarian Schmidtea mediterranea. Cell Stem Cell *3*, 327-339.

Guo, T., Peters, A.H., and Newmark, P.A. (2006). A Bruno-like gene is required for stem cell maintenance in planarians. Dev Cell *11*, 159-169.

Gurley, K.A., Rink, J.C., and Sanchez Alvarado, A. (2008). Beta-catenin defines head versus tail identity during planarian regeneration and homeostasis. Science *319*, 323-327.

Lapan, S.W., and Reddien, P.W. (2012). Transcriptome analysis of the planarian eye identifies ovo as a specific regulator of eye regeneration. Cell Rep *2*, 294-307.

Orii, H., Sakurai, T., and Watanabe, K. (2005). Distribution of the stem cells (neoblasts) in the planarian Dugesia japonica. Dev Genes Evol *215*, 143-157.

Reddien, P.W., Oviedo, N.J., Jennings, J.R., Jenkin, J.C., and Sanchez Alvarado, A. (2005). SMEDWI-2 is a PIWI-like protein that regulates planarian stem cells. Science *310*, 1327-1330.

Rink, J.C., Vu, H.T., and Sanchez Alvarado, A. (2011). The maintenance and regeneration of the planarian excretory system are regulated by EGFR signaling. Development *138*, 3769-3780.

Robb, S.M., Ross, E., and Sanchez Alvarado, A. (2008). SmedGD: the Schmidtea mediterranea genome database. Nucleic Acids Res *36*, D599-606.

Shin, J.T., Priest, J.R., Ovcharenko, I., Ronco, A., Moore, R.K., Burns, C.G., and MacRae, C.A. (2005). Human-zebrafish non-coding conserved elements act in vivo to regulate transcription. Nucleic Acids Res *33*, 5437-5445.

Witchley, J.N., Mayer, M., Wagner, D.E., Owen, J.H., and Reddien, P.W. (2013). Muscle cells provide instructions for planarian regeneration. Cell Rep *4*, 633-641.

Yelon, D., Horne, S.A., and Stainier, D.Y. (1999). Restricted expression of cardiac myosin genes reveals regulated aspects of heart tube assembly in zebrafish. Dev Biol *214*, 23-37.
